# The Effect of a Fluorophore Photo-Physics on the Lipid Vesicle Diffusion Coefficient Studied by Fluorescence Correlation Spectroscopy

**DOI:** 10.1007/s10895-015-1752-5

**Published:** 2015-12-22

**Authors:** Dominik Drabik, Magda Przybyło, Aleksander Sikorski, Marek Langner

**Affiliations:** Laboratory for Biophysics of Macromolecular Aggregates, Department of Biomedical Engineering, Wroclaw University of Technology, Pl. Grunwaldzki 13, 50-377 Wroclaw, Poland; Lipid Systems sp. z. o. o. [Ltd], ul. Duńska 9, 54-066 Wrocław, Poland; Laboratory of Cytobiochemistry, Faculty of Biotechnology, University of Wroclaw, ul. Joliot-Curie 14a, Wrocław, Poland

**Keywords:** Fluorescence correlation spectroscopy, Dynamic light scattering, Photo-bleaching, Blinking, Fluorescent probes

## Abstract

Fluorescence Correlation Spectroscopy (FCS) is a technique, which allows determination of the diffusion coefficient and concentration of fluorescent objects suspended in the solution. The measured parameter is the fluctuation of the fluorescence signal emitted by diffusing molecules. When 100 nm DOPC vesicles labeled with various fluorescent dyes (Fluorescein-PE, NBD-PE, Atto488 DOPE or βBodipy FL) were measured, different values of diffusion coefficients have been obtained. These diffusion coefficients were different from the expected values measured using the dynamic light scattering method (DLS). The FCS was initially developed for solutions containing small fluorescent molecules therefore the observed inconsistency may result from the nature of vesicle suspension itself. The duration of the fluorescence signal may depend on the following factors: the exposure time of the labeled object to the excitation beam, the photo-physical properties (e.g., stability) of a fluorophore, the theoretical model used for the calculations of the diffusion coefficient and optical properties of the vesicle suspension. The diffusion coefficients determined for differently labeled liposomes show that its dependence on vesicle size and quantity of fluorescent probed used for labeling was significant demonstrating that the fluorescence properties of the fluorophore itself (bleaching and/or blinking) were critical factors for a correct outcome of FCS experiment. The new, based on combined FCS and DLS measurements, method for the determination of the focal volume prove itself to be useful for the evaluation of a fluorescence dye with respect to its applicability for FCS experiment.

**Electronic supplementary material:**

The online version of this article (doi:10.1007/s10895-015-1752-5) contains supplementary material, which is available to authorized users.

## Introduction

Fluorescence Correlation Spectroscopy (FCS) is a well-established technique that allows measuring diffusion coefficients and chemical reaction rates in solutions [1]. The technique is based on the measurements of the fluctuations of fluorescence intensity emitted from the defined volume of the sample. These fluctuations may be produced by fluorescent molecules diffusing in and out of a sampling volume, which is defined by laser focal volume, and/or photochemical reaction involving fluorescent molecules within the focal volume [2]. The focal volume is therefore a central technical parameter of the FCS method. To evaluate correctly the dynamics of the sample it is critical to measure accurately the size of the focal volume. There are three approaches used to determine the size of the focal volume; using samples containing molecules at known concentration, measuring solution with molecules of predetermined diffusion coefficient or scanning the static conformational feature of the sample [3]. All three methods require independent calibration system, which physical, chemical or optical properties may differ from that of the sample. For example the water soluble fluorescence probes (Alexa or Rhodamine) are typically used for the calibration purposes [4]. The diffusion coefficients of such probes, assume to be known in advance, may very due to the nature of the solution containing the fluorescent probe and/or measuring technique used. This in turn will affect the determined value of the focal volume. The other difficulty may result from the differences between a sample and the calibration system with the respect to their optical properties. Typically, the focal volume is determined experimentally using diluted solution of hydrophilic fluorescent dye. The resulting value of the focal volume is derived from experimental data using a series of methodological and technical assumptions regarding mainly the geometry of the optical system and illumination beam, probe stability and negligible influence of other optical effects [5]. Whereas the determination of the focal volume, based on the diffusion of the small fluorescent dye, can be readily used for the characterization of processes taking place in solutions they may not suffice when suspensions of particulate systems are studied [6–9]. The quantitative analysis of processes taking place in a suspension may be affected by slow diffusion time of particulate, photo-physical properties of a fluorescence dye and scattered light. In the paper a methods for the determination of the focal volume and the evaluation of the photo-bleaching and/or blinking effect on FCS measurements are presented. The presented approach relies on the combination of the two autocorrelation techniques; dynamic light scattering (DSC) and fluorescence correlation spectroscopy (FCS). The method makes possible the measurement of the correct value of the diffusion coefficient and the determination of the focal volume “in situ”, in the suspension of particulates labelled with fluorescent probe.

## Materials and Methods

### Chemicals

1,2-Dioleoyl-sn-glycero-3-phosphocholine (DOPC) and fluorescent dyes: Fluorescein-PE (1,2-dioleoyl-*sn*-glycero-3-phosphoethanolamine-N-(carboxyfluorescein) (ammonium salt)), Rhodamine-PE (1,2-dioleoyl-*sn*-glycero-3-phosphoethanolamine-N-(lissamine rhodamine B sulfonyl) (ammonium salt)) and NBD-PE (*N*-(7-Nitrobenz-2-Oxa-1,3-Diazol-4-yl)-1,2-Dihexadecanoyl-*sn*-Glycero-3-Phosphoethanolamine, (triethylammonium salt)) were purchased from Avanti Polar Lipids, Inc. (Alabaster, AL). Atto488 DOPE (1,2-dioleoyl-*sn*-glycero-3-phosphoethanolamine) was purchased from Atto-Tec (Germany) whereas βBodipy FL (2-(4,4-difluoro-5,7-diphenyl-4-bora-3a,4a-diaza-*s*-indacene-3-pentanoyl)-1-hexadecanoyl-*sn*-glycero-3-phosphocholine) and Alexa488 were purchased from Life Technologies (USA). NaHPO_4_ and Na_2_H_2_PO_4_ salts were purchased from POCH (Poland). Aqueous solutions were prepared with Milli-Q (18 MOhm) water quality and passed through 0.45 μm pores filter in order to remove any physical impurities.

### Preparation of Fluorescently Labelled Liposomes

The large unilamellar vesicles (LUVs) containing selected fluorescent dye were formed using method described previously [10]. In short, appropriate amounts of lipid dissolved in chloroform (100 mg/ml) was mixed with the fluorescent dye. The organic solvent was removed under the stream of nitrogen followed by the overnight incubation under vacuum. The resulting dry lipid film was then hydrated with phosphate buffer solution and vortexed to obtain a milky suspension. The resulting multilamellar liposomes were extruded through the polycarbonate filter with 100 nm or 50 nm pores (Whatman, Germany). The final lipid concentration was 50 μM and the quality of the liposome suspension was tested prior and after each measurement using the DLS technique (NanoSizer ZS, Malvern, UK).

### Fluorescence Correlation Spectroscopy Measurements and Data Analysis

FCS measurements were carried out on a Carl Zeiss Confocor 3 combied with MicroTime 200 inverted epifluorescence confocal microscope (Carl Zeiss, Picoquant, Germany). Optical configuration consists of a pulsed diode laser (LDH-P-C- 470, 470 nm, Picoquant, and LDH-DC-635, 635 nm, Picoquant) producing 80 ps pulses at a 40 MHz repetition rate, a filter set (clean up filter 470/20, dichroic mirror 505DRLP and long-pass filter 510) (Omega Optical), and a water-immersed objective (1.2 NA, 40x) (Olympus). Low power (5 *μ*W) at the back aperture of the objective was chosen to minimize the photobleaching and saturation effects. The series of fluorescence events were autocorrelated using SymPhoTime software with number of adjacent τ values set to be equal 16. For each measurement the fluorescence lifetime of a fluorophore was determined (PicoQuant SymPhoTime 5.3). The obtained autocorrelation curves were fitted with both standard three dimensional model of free diffusion (Brownian motion model) and Liposome model developed by Bogaart et al.[11], which assumes the Maxwell distribution of vesicle sizes and accounts for the effect of vesicle sizes on the calculated concentration of liposomes. The Brownian motion model for a single population of vesicles is described by Eq. 1, where parameter *N* is defined as an average number of fluorescent objects passing the focal volume, *κ* is a length to diameter ratio of the focal volume defined by Eq. 2. In Eq. 2 z_0_ and w_xy_ are the focal radius along the optical axis at $$ \raisebox{1ex}{$1$}\!\left/ \!\raisebox{-1ex}{${e}^2$}\right. $$ intensity and the lateral focal radius at $$ \raisebox{1ex}{$1$}\!\left/ \!\raisebox{-1ex}{${e}^2$}\right. $$ intensity expressed in microns, respectively. Those values were used to define focal volume described by the Eq. 3. The concentration of fluorescent objects in the sample was calculated using Eq. 4 (where N_A_ is the Avogadro number) and the correlation between the diffusion coefficient and the diffusion time is described by the Eq. 5.

1 $$ G\left(\tau \right)=\frac{1}{N}{\left(1+\frac{\tau }{\tau_D}\right)}^{-1}{\left(1+\frac{\tau }{\tau_D{\kappa}^2}\right)}^{-1/2} $$

2 $$ \kappa =\frac{z_0}{w_{xy}} $$

3 $$ {V}_f={\pi}^{\frac{3}{2}}\cdot {w}_{xy}^2\cdot {z}_0 $$

4 $$ C=\frac{N}{V_f\cdot {N}_A} $$

5 $$ D=\frac{\omega_{xy}}{4{\tau}_D} $$

6 $$ G\left(\tau \right)=\frac{1}{N}\cdot {\left(1+\frac{4\cdot D\cdot \tau }{\omega_{xy}^2}\right)}^{-1}\cdot {\left(1+\frac{4\cdot D\cdot \tau }{\kappa \cdot {\omega}_{xy}^2}\right)}^{-1/2} $$

The main objective of presented procedure is to determine the focal volume based on the predetermined value of the diffusion coefficient. This is achieved by fitting experimentally determined autocorrelation function to the Eq. 6. The model described by Eqs. 1 and 6 treats liposomes as uniform population of small fluorescence objects. However, as demonstrated by result of dynamic light scattering measurements the sizes of liposomes are presented as a population with the average and standard deviation values. To account for the statistical character of the sample the model proposed by Bogaart et al. (1) has been implemented. In this model the liposome sizes are represented by the Maxwell distribution (approximated with normal distribution) parameterized using the average radius “R” and the standard deviation “a”. The fraction of liposomes with radius r is given by Eq. 7, where erf(*x*) is the “error function”.

7 $$ k(r)=\frac{2{e}^{-\frac{{\left(r-R\right)}^2}{a^2}}}{a\sqrt{\pi}\left(1+\mathrm{e}\mathrm{r}\mathrm{f}\left(\frac{R}{a}\right)\right)} $$

Since sizes of individual liposomes are different, therefore the number of dyes per liposome (W(r)) is also different as demonstrated by Eq. 8 where c represents the dye to lipid molar ratio and A is the area per lipid molecule in nm^2^. The diffusion coefficient D of liposome depends on its radius according to Einstein-Stokes relationship (Eq. 9), where γ is a constant dependent on the viscosity and temperature of the medium. Fluorescence autocorrelation function of liposomes suspension is given by Eq. 10, where C_l_ is the concentration of liposomes (fluorescent objects).

8 $$ W(r)=\frac{8\pi c}{A}{r}^2 $$

9 $$ D(r)=\frac{\gamma }{r} $$

10 $$ G\left(\tau \right)=\frac{1}{V_{eff}\cdot {C}_l}\cdot \frac{{\displaystyle {\int}_0^{\infty }}W{(r)}^2\cdot k(r)\cdot {\left(1+\frac{4\tau D(r)}{w_{xy}^2}\right)}^{-1}\cdot {\left(1+\frac{4\tau D(r)}{z_0^2}\right)}^{-\frac{1}{2}}dr}{{\left({\displaystyle {\int}_0^{\infty }}W(r)k(r)dr\right)}^2} $$

The autocorrelation curve (Eq. 10) combined with Eqs. 7, 8 and 9 results with the Eq. 11.

11 $$ G\left(\tau \right)=\frac{{\displaystyle {\int}_0^{\infty }}2{r}^2\cdot {e}^{\left(\frac{{\left(r-R\right)}^2}{a^2}\right)}\cdot {\left(1+4\tau \frac{\gamma }{r}\cdot {\left(\frac{V_{eff}}{\pi^{\frac{3}{2}}\cdot \kappa}\right)}^{-\frac{2}{3}}\right)}^{-1}\cdot {\left(1+4\tau \frac{\gamma }{r}\cdot \frac{1}{\kappa}\cdot {\left(\frac{V_{eff}}{\pi^{\frac{3}{2}}\cdot \kappa}\right)}^{-\frac{2}{3}}\right)}^{-1/2}dr}{\left({V}_{eff}{C}_l\right)\cdot \left(\frac{8\pi c}{A}\right)\cdot {\left(a\sqrt{\pi}\left(1+\mathrm{e}\mathrm{r}\mathrm{f}\left(\frac{R}{a}\right)\right)\right)}^{-1}\cdot {\left({\displaystyle \underset{0}{\overset{\infty }{\int }}}2{r}^2\cdot {e}^{\left(\frac{{\left(r-R\right)}^2}{a^2}\right)}dr\right)}^2} $$

The numerators of the Eq. 11 cannot be derived analytically therefore it was approximated numerically using Simpson’s method. It has been demonstrated that eight steps within the range from *r* = *R* − 2*a* to *r* = *R* + 2*a* were sufficient (there have been no significant improvement of calculated parameters when higher number of steps have been used). The Eq. 11 was fitted into autocorrelation curves using both Levenberg-Marquardt and genetic algorithms. Levenberg-Marquardt and genetic algorithms were implemented using OriginLab Origin 9.0 and Matlab 7 (Optimization Toolbox with population size of 200, roulette selection of next generation, 200 generations and single point crossover), respectively.

For each sample 50 measurements were performed. In the fitting procedure, instead of a specific diffusion coefficient, the range differing by 5 % in each direction from the average value were enforced. Using Brownian model it was possible to determine the concentration of liposome and the focal volume. When the non-homogeneous liposome population model was used the concentration, averaged vesicle radius, the standard deviation and the focal volume were determined.

### The Determination of the Focal Volume Using the Predetermined Liposome Diffusion Coefficient

The determination of the microscope focal volume is based on the assumption that the fluorescence of a small water-soluble fluorophore is sufficiently stable to ensure continuous fluorescence emission when the molecule is present in the focal volume. This condition may not be satisfied in all experimental conditions leading to erroneous assumptions as has been demonstrated for Rhodamine 6G dye [12]. We proposed new liposome-based method for the determination of the focal volume. The method accounts for effects specific for suspensions of particulates and not accounted for by a model composed of identical fluorescent molecules. Vesicle suspension is a statistical entity where individual objects differ in size, number of fluorescent probes or topological alterations due to thermal fluctuations or compositional heterogeneity. The method relies on the diffusion coefficient of vesicles predetermined using dynamic light scattering method. The diffusion coefficient is available in the form of the average value or as a distribution. In order to account for the statistical nature of the particulate suspension a statistically relevant number of measurements need to be performed (50 in experiments used for calculation presented in the paper). The value of the focal volume is calculated from each individual autocorrelation curve and the statistical distribution of obtained values is constructed. In all cases the normal distribution of the focal volume was obtained. The resulted distributions were fitted with Gaussian function using Levenberg-Marquardt and genetic algorithms to determine the mean size and the variance of the focal volumes.

## Results and Discussion

**Fig. 1:**
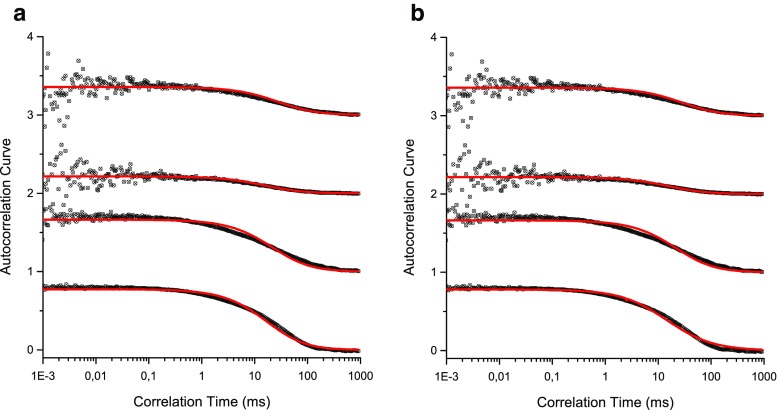
Autocorrelation functions collected for samples containing liposomes labelled with different fluorescent probes and fitted to **a** Brownian motion model and **b** Liposome model using Levenberg-Marquardt algorithm. From top to bottom: autocorrelation curves obtained for vesicles labelled with NBD, Fluorescein, βBodipy or Atto dyes

**Fig. 2:**
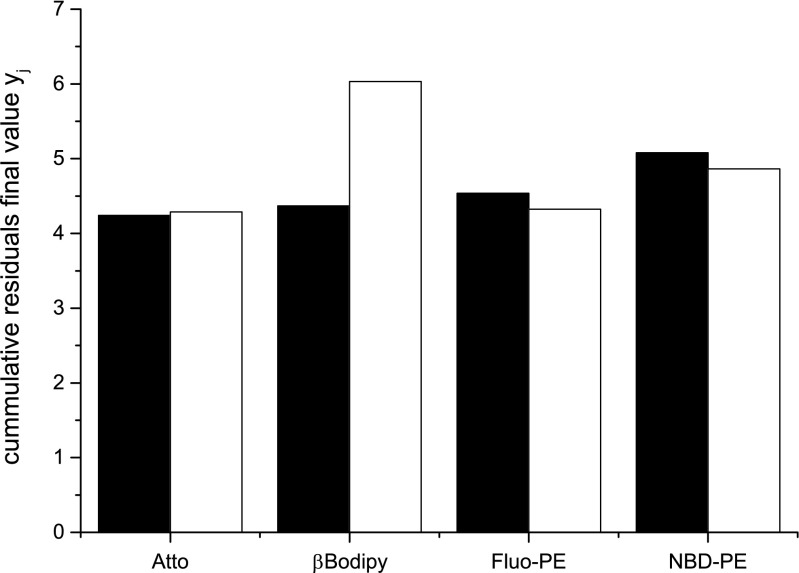
The final value of cumulative residues between experimental data and fitted curves. Each value of the cumulative residue is the average of 5 measurements. Black bars represent cumulative residuals obtained for Levenberg-Marquardt algorithm used for fitting while white bars represent data obtained for genetic algorithms. The correlation functions were collected for samples containing 100 nm liposomes with the fluorescent dye to lipid molar ratio equals to 1:500

In order to reduce the possible errors of the liposome diffusion coefficient evaluation its value was measured using dynamic light scattering (DLS). The average vesicle sizes was equal to 120 nm ± 10 nm and 70 nm ± 10 nm when filter with pore diameter of 100 nm and 50 nm were used, respectively. In all cases the polydispersity index was smaller than 0.1. The dynamic light scattering is free of artefacts typically accompanying fluorescence measurement and it requires limited assumptions regarding the liposome suspension. FCS measurement, on the other hand, is based on a series of assumptions needed for the construction of the three-dimensional model of free diffusing, not interacting, spherical objects having same sizes and masses [7]. In reality suspension of liposomes contains vesicle of different sizes and shapes requiring different theoretical approach [11]. The other factor affecting the outcome of the FCS experiment is the photo-stability of the fluorescent moiety. Whereas fluorescence decay of solution containing the water-soluble fluorescence dye caused by photobleaching can be accurately characterized, the fluorescence decay for liposome labelled with fluorescent dye is not [7, 13, 14]. This is because properties of fluorescent probes are different when associated with the lipid membrane [15, 16]. In addition, the fluorescence emitted by vesicle slowly passing through the focal volume is produced collectively by a number of probes associated with the membrane. The combination of a long exposure time leading to extensive bleaching, ill define subpopulations of fluorescent probes, non-uniform excitation beam and the presence of scatter light result with difficult to anticipate fluorescent events accompanying the vesicle passage through the focal volume. This will affect the shape of the autocorrelation function and consequently interfere with the determined value of the diffusion coefficient. Figure 1 shows autocorrelation functions determine for 100 nm vesicles labelled with different fluorescent dyes. The experimental data were fitted with curves derived from Brownian and Liposome models using both Levenberg-Marquardt and genetic algorithms. The quality of fitting was evaluated using the plot of residuals traces and the plot of cumulative residuals *y*_*j*_ = ∑_*i* = 0_^*j*^|*dy*_*i*_|. The Figure 2 shows that the quality of fitting is independent on the fitting procedure and physical model selected. Time evolution of the parameter is presented if figure S.1.2 in Supplementary Information. Therefore the data analysis process can be optimized from the technical point of view. Since the genetic algorithm calculations are time-expensive, Levenberg-Marquardt method can be safely used to handle data obtained in FCS measurements of liposome suspensions.

**Fig. 3:**
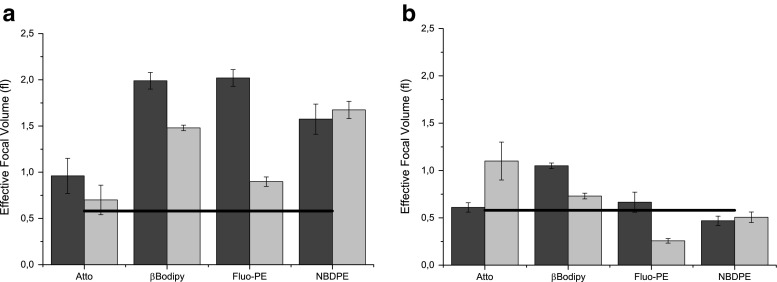
Values of diffusion coefficients obtained using FCS technique for 100 nm (**a**) and 50 nm (**b**) liposomes. Values determined using DLS technique are indicated with *thick black lines*

In most cases the quality of fitting did not change regardless of the model (Brownian or liposomal) used. Therefore the first model was used in all subsequent calculations. The other sources of errors may result from the chemical instability of fluorophore and/or sample optical properties altered by the presence of liposomes [6]. In a typical fluorescence correlation spectroscopy experiment the focal volume is determined using the water-soluble dye such as Rhodamine or Alexa 488 [17]. In this approach it is assumed that the water-soluble fluorescence dye diffusion coefficient is known in advance and that the photo-bleaching effect is negligible. In the paper the Alexa488 dissolved in water was used to determine the focal volume according to the well-established calibration procedure [3]. Based on the literature data it has been assumed that the diffusion coefficient equals to 452,6 μm^2^/s in 24C^o^ [18]. The determined value of the focal volume equals to *V*_*f*_ = (0.58 ± 0.06)*fl* (Figure S.1 in Supplementary Materials shows autocorrelation curves collected during the calibration experiment). Figure 3 shows diffusion coefficients determined for a series of vesicle suspensions labelled with different fluorescence probes (at concentrations equal to 0,5 mol %). The value of the focal volume was determined in separate experiment using water-soluble Alexa 488 dye. The diffusion coefficient of vesicles measured with dynamic light scattering is shown for comparison.

**Fig. 4:**
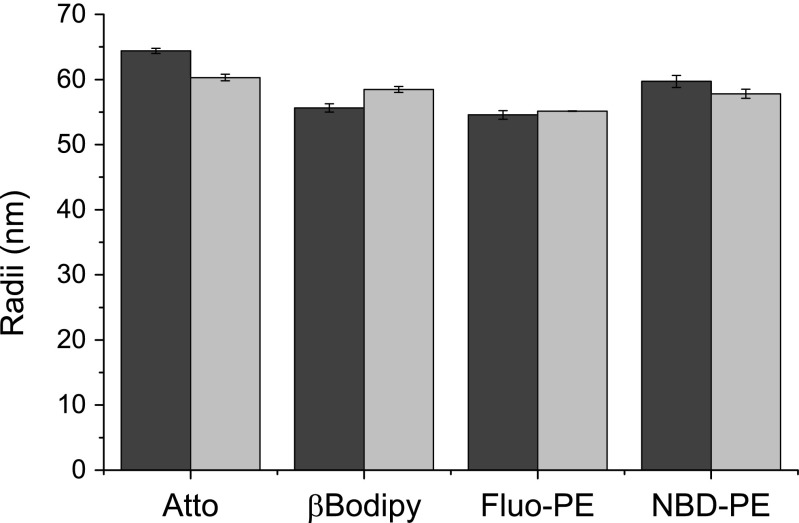
The effect of fluorescence probe type and quantity on liposome sizes determined with the dynamic light scattering method. Liposomes were extruded through the membrane with 100 nm pores. The dye:lipid ratios were equal to 1:500 (*black bars*) and 1:1000 (*light bars*)

The value of the diffusion coefficient determined with the dynamic light scattering technique was assumed to be a correct one since the DLS measurement is free of artefacts specific for fluorescence based methods. Data presented on Figure 3 shows that the determined diffusion coefficients depend on the type of fluorescence probe and the size of vesicle used. The difference between values of the diffusion coefficients determined with FCS technique and the expected ones is more pronounced for smaller vesicles. For example the determined value of the average size differs by more than 100 % when vesicles are labelled with βBodipy (Fig. 3b). Similarly large difference was observed for vesicles labelled with Atto probe. Surprisingly, the discrepancy was less significant when vesicles were labelled with fluorescein-PE or NBD-PE dyes. The differences are less pronounced for larger vesicles (extruded using 100 nm membrane). There are four possible explanations for the observed differences; 1) the presence of fluorescent probe changes the vesicle size; 2) the location (fluorescence) of fluorophore depends on the vesicle curvature; 3) the fluorophore is bleached when passing the microscope focal volume or 4) the fluorophore intrinsic propensity for blinking influences the fluorescent emission. To exclude the first possibility liposomes labelled with different probes were measured using the dynamic light scattering technique. Figure 4 shows the effect of different fluorescent probes on sizes of vesicles extruded through membranes with 100 nm pores.

**Fig. 5:**
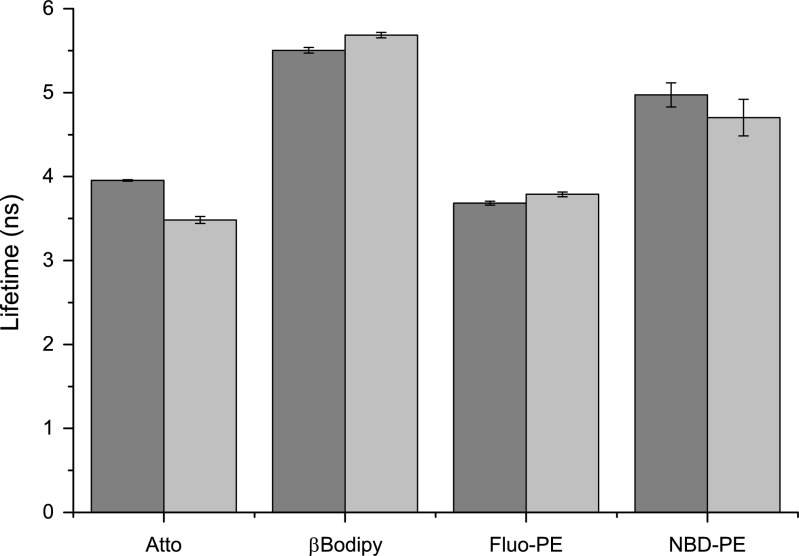
Fluorescence lifetimes of fluorescent probes incorporated into vesicle differing in sizes. *Darker* and *lighter bars* represent results for 100 nm and 50 nm vesicles, respectively

Data presented in Fig. 4 demonstrates that the presence of any fluorescence probe in quantities used in the course of presented studies did not affect the size of liposome. The other possible source of the alteration of the fluorescence emission intensity is a changed in a polarity of a fluorophore surrounding, which can be evaluated with the fluorescence lifetime of the fluorophore [19]. Such effect is likely to occur when the vesicle differ with regard to their curvature. Figure 5 shows lifetimes of fluorophores used in vesicle extruded through 100 nm and 50 nm membranes.

**Fig. 6:**
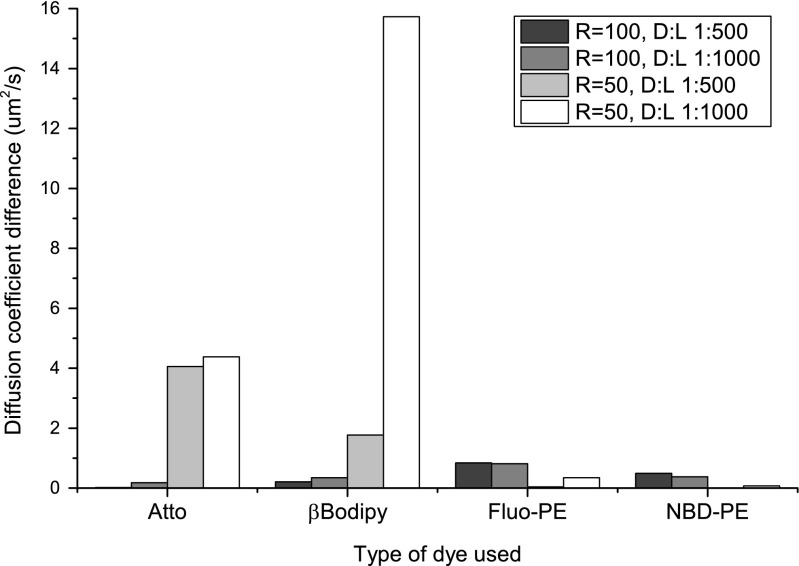
The effect of fluorescent probe quantity on the difference between values of the diffusion coefficients measured using FCS technique and that determined with dynamic light scattering method

Figure 5 shows that for vesicles containing βBodipy and Fluo-PE there is no effect of vesicle size on the fluorescence lifetime of the fluorophore. The effect is noticeable for vesicle containing Atto and NBD-PE dyes. The changing vesicle curvature should not alter the fluorescence lifetime of βBodipy and Fluo-PE, because they are located in hydrophobic membrane interior and in the aqueous phase adjacent to the lipid membrane, respectively [20, 21]. The other probes NBD and Atto are located within the membrane interface, therefore the polarity of probes surroundings will depend on the membrane curvature affecting their fluorescence properties [22, 23]. The fluorescence lifetimes of Atto and NBD fluorophores are smaller when measured in vesicles extruded through 50 nm pores. The determined values of fluorescence lifetimes are in good agreement with the available literature data for Fluorescein and βBodipy FL in water [19], NBD-PE in DMPC lipid bilayer [24] and manufacturer information for Atto488 DOPE It can be concluded so far that vesicles are of the same sizes, regardless on type and quantities of fluorescence probe used, and immediate environment of all fluorophores does not depend significantly on the vesicle size therefore the only reason for observed variations in values of determined diffusion coefficient is the performance of each fluorophore when continuously illuminated. There are two possible processes causing the effect; photobleaching and/or blinking [25–28]. The photo-bleaching is a process leading to chemical alteration of the fluorophore causing the permanent loss of its capability to emit fluorescence whereas blinking is associated with the temporary loss of capacity to fluoresce. The blinking off time may range from milliseconds up-to seconds, depending on the type of the fluorophore. Therefore the blinking time-scale is similar to the time of fluorescent object crossing the focal volume of the excitation beam. Data presented in Fig. 3 shows that the discrepancies in values of vesicle diffusion coefficients are more pronounced for small vesicles. The diffusion coefficients do not differ so much for vesicles extruded through 100 nm membranes. The photo-bleaching will result with the shortening of the fluorescence signal causing the overestimation of diffusion coefficient values. Invariance of the diffusion coefficient of larger vesicles on a fluorophore used shows that the photobleaching is not an important factor. However, the large variations in values of the diffusion coefficient for smaller vesicles show that when number of fluorescent probes decreases from about 200 and 100 (depending on lipid to dye ratio), for larger vesicles, to about 50 and 25, for smaller vesicles, the effect of fluorescent probe become significant. The small number of fluorescent molecules present in small vesicles would result with conditions close to a single molecule limit making the blinking effect significant. This is supported by the dependence of the diffusion coefficient on the quantity of fluorophores present in vesicles. The reduction of the number of fluorescent probes caused the elevated difference of the determined value of the diffusion coefficient and that determined using the dynamic light scattering is clearly visible in Fig. 6.

### The Method for the Determination of the Focal Volume in a Suspension of Vesicles

**Fig. 7:**
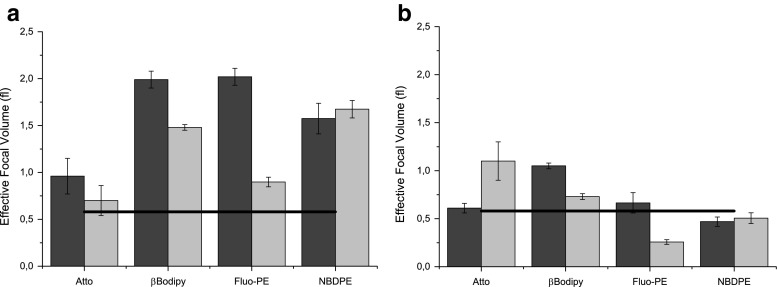
Focal volumes calculated by assuming parameters acquired from DLS measurements using Levenberg-Marquardt algorithm to fit experimental data to **a** Brownian model and **b** liposome model. The black line represents value of the focal volume obtained by the calibration method using water soluble Alexa dye. The dye:lipid ratios were equal to 1:500 (*black bars*) and 1:1000 (*light bars*)

The traditional experimental determination of the focal volume does not account for the statistical nature of suspension of particulates. Vesicles differ in sizes, number of incorporated fluorescent probes in a single vesicle, their topology and events related to fluorophores themselves such as photo-bleaching or blinking. Therefore the statistical approach should be more appropriate for this type of samples. Specific histograms of selected samples are presented in Supplementary Information. Figure 7 presents values of focal volumes determined by fitting Brownian and liposome models to experimental data using Levenberg-Marquardt algorithm. Results for genetic algorithms are presented in figure S.7 and S.8 in Supplementary Information.

**Fig. 8:**
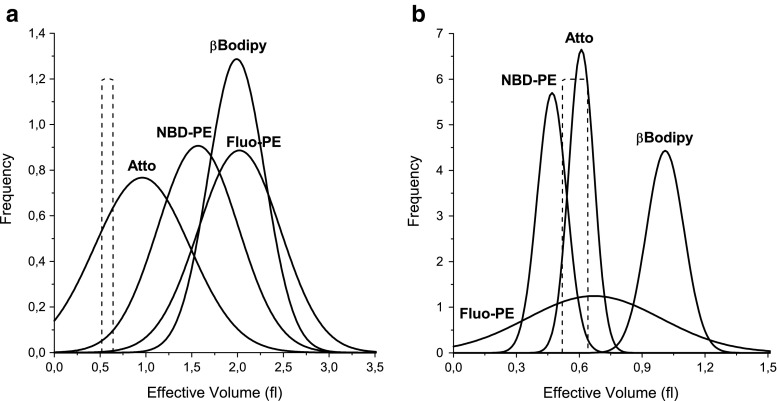
Comparison of distributions of focal volumes determined with liposomes labeled with different fluorescent dyes using diffusion coefficient determined in DLS experiment. Presented data have been obtained for 100 nm liposome suspensions labeled with 1:500 dye:lipid ratio using **a** Brownian and **b** Liposome models fitted with Levenberg-Marquardt algorithm. *Dashed lines* represent results from calibration procedure based on the Alexa dye solution

In Fig. 7 values of focal volumes are represented by the peak value (μ) of the distribution while uncertainty is represented by the standard deviation σ of the normal distribution. It can be therefore observed that, when statistical approach is used, the determined focal volume depends on an experimental system and is different from that measure with the small molecular fluorophore (Alexa). The difference is more pronounced when the 3D Brownian model is employed (Fig. 7a). The differences are mitigated when the non-uniformity of vesicle population is accounted for by applying the liposome model (Fig. 7b). The reduced difference between the focal volumes determined using the vesicle-based measurement and Alexa-based measurement shows that the effect of dye photostability is substantially reduced. The difference between two models is demonstrated by distributions of focal volumes (Fig. 8). While for both models fitting qualities are similar, the Liposome model provides more accurate values for all dyes except Fluo-PE. The wide distribution of the focal volume determined for Fluo-PE probe can be explained by the fact, that measurements were performed in pH 7 where the fluorescence intensity is low and the protonation and deprotonation of the probe may interfere with the measurement.

## Conclusions

The fluorescence correlation spectroscopy (FCS) is routinely used to measure the diffusion coefficient of various molecules and their ensembles as well as its alteration by association/dissociation processes. The determination of the diffusion coefficient requires the knowledge of the predetermined focal volume, a parameter specific for the particular experimental set-up. This is usually done with small water-soluble fluorescent molecule. Next, the diffusion coefficient is determined from fluorescence impulses generated by molecules passing the illuminated volume. When larger entities are studied, as it is the case for lipid vesicles, a number of effects, specific for such experimental system, need to be accounted for. Firstly, the population of lipid vesicle is usually not uniform what may require dedicated data analysis algorithms. Secondly, the presence of particulates changes optical parameters of the sample by adding the light scattering effect. Thirdly, the fluorescence properties of fluorescent dye may change due to the topological alterations of particulates. Fourthly, the low diffusion coefficient of large supramolecular ensembles result with the extended exposure of fluorophores to high intensity illumination what may altered probe stability. In order to validate fluorescence correlation spectroscopy method the determined diffusion coefficient were compared to that obtained using dynamic light scattering method. In the course of presented data it has been established that the value of the diffusion coefficient of lipid vesicles is invariant on the algorithm used. There was also no significant difference between values of the diffusion coefficients calculated using biophysical models based on the assumption of uniform and non-uniform vesicle distributions. The light scattered by vesicles did not interfere with determined value of the diffusion coefficient. However, it has been observed that the effect of vesicle size on the measure diffusion coefficient depends on a type of fluorescent probe used. This cannot be explained by altered fluorescence properties of fluorophores, since there has been no effect of vesicle size on the value of the fluorescence lifetime when fluorophores located in well-defined environment were used and only small effect when vesicles were labelled with an interfacial dyes. It has been concluded that, the determined value of the diffusion coefficient is affected the most by the fluorophore stability upon illumination. Fluorescein-PE and NBD-PE were performing well whereas Atto and βBodipy produced erroneous values of the diffusion coefficient. The effect is likely a result of a probe blinking as demonstrated by the dependence of the discrepancy between measured and expected values of the diffusion coefficients on number of probes present in a single vesicle. When the focal volume is determined from FCS experiment using the diffusion coefficient derived from dynamic light scattering experiment the differences between models assuming uniform and non-uniform vesicle size distribution become significant.

## Electronic supplementary material

ESM 1 (DOCX 18334 kb)
