# Supplementary material for: The Effect of a Fluorophore Photo-Physics on the Lipid Vesicle Diffusion Coefficient Studied by Fluorescence Correlation Spectroscopy
Source: J Fluoresc. 2015 Dec 22;26:661–9. doi: 10.1007/s10895-015-1752-5 (PMC4773467; doi:10.1007/s10895-015-1752-5)
Supplement: Supplementary file 1 — (DOCX 18334 kb) [file 10895_2015_1752_MOESM1_ESM.docx]

**The effect of a fluorophore photo-physics on the lipid vesicle diffusion coefficient studied by Fluorescence Correlation Spectroscopy**

**SUPPLEMENTARY INFORMATION**

Dominik Drabik^1^, Magda Przybyło^1,2^, Aleksander Sikorski^3^, Marek Langner^1^

^1^ Laboratory for Biophysics of Macromolecular Aggregates, Department of Biomedical Engineering, Wroclaw University of Technology, Pl. Grunwaldzki 13, Wroclaw, Poland

^2^ Lipid Systems sp. z. o. o. [Ltd], ul. Duńska 9, Wrocław, Poland

^3^ Laboratory of Cytobiochemistry, Faculty of Biotechnology, University of Wroclaw, ul. Joliot-Curie 14a, 50-383 Wrocław, Poland

# The focal volume determination using Alexa fluorescent dye and fitting methods testing.

| 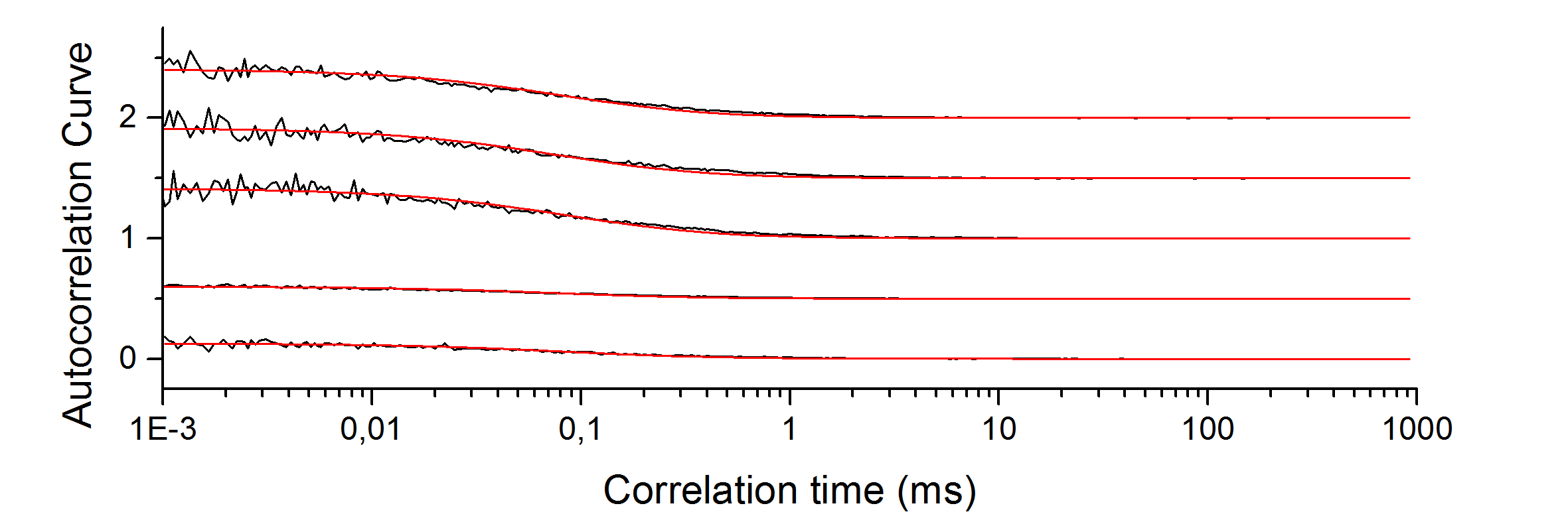 |
| --- |
| Figure S.1.1. Examples of recorded autocorrelation curves of Alexa488 dye dissolved in water. From down to top measurements were performed for 60, 300, 300, 300 and 600 s. |

| 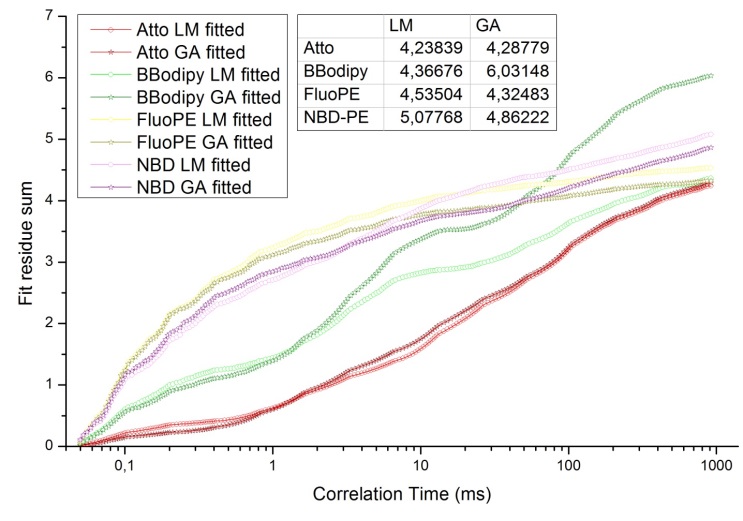 |
| --- |
| Figure S.1.2. The plot of the fractional sum of residues of the fit as a function of the correlation time. Each value of the residue is the average of 5 measurements. The correlation function was collected for 100 nm liposomes with the fluorescent dye to lipid ratio equals to 1:500. |

The focal volume was calculated from 5 measurements using Alexa488 dye dissolved in water. Three measurements were carried out for 300s, one for 60s and one for 600s. The determined focal volume is the average value over all measurements. The determined focal volume equals to $V_{eff}=0,58\pm0,06 fl$. Figure S.1 shows recorded autocorrelation curves along with the fit to the three-dimensional model. The measurements were performed with attenuator in the position 1.

Two methods of fitting the models to experimental data were tested; Levenberg-Marquardt and genetic algorithms. Figure S.1.2. shows the fractional sum of residual values $y_{j}$ as a function of the correlation time.

# FCS measurements

Sample was measured 50 times in order to collect statistically relevant data. For each sample a set of parameters have been determined and summarized in the Table. The following quantities are presented: diffusion constant, concentration of liposomes, the averaged vesicle radius, distribution of sizes (Liposome model), the focal volume along with the histogram. Each sample description contains the fluorescence dye used, the pore size in polycarbonate filter used for vesicle formation and the molar ratio of fluorescence probes and lipids. Liposome concentration in Brownian model is calculated according to equation S.1 and its uncertainty according to equation S.2. Each determined focal volume is included in the accompanying histogram. Each sample is analyzed using both Levenberg-Marquardt algorithm (LM) or genetic algorithm (GA) for fitting.

$C=\frac{\left\langle N \right\rangle}{N_{a}\cdot V_{eff}}$ (S.1)

$\Delta C=\frac{\Delta\left\langle N \right\rangle}{N_{a}\cdot V_{eff}}+\frac{\Delta V_{eff}\cdot\left\langle N \right\rangle}{\left( N_{a}\cdot V_{eff} \right)^{2}}$ (S.2)

Atto488-PE; D=100nm; ratio 1:500

| Technique, model, fitting algorithm | Diffusion [um/s^2^] | Radius of vesicle [nm] | Vesicles spread [nm] | Liposome Concentration [nM] | Focal Volume [fl] | Corresponding figure number |
| --- | --- | --- | --- | --- | --- | --- |
| DLS | 3,80 ± 0,02 | 62,4 ± 0,4 | 22,5 ± 2,2 | - | - | - |
| FCS, Brownian model, LM algorithm | 3,94 ± 0,14 | - | - | 1,7 ± 0,2 | 0,96 ± 0,19 | S.2.1 |
| FCS, Liposome model, LM algorithm | 3,91 ± 0,28 | 61,5 ± 4,6 | 23 ± 9 | 4,1 ± 0,9 | 0,61 ± 0,05 | S.2.2 |
| FCS, Brownian model, Genetic algorithm | 3,86 ± 0,12 | - | - | 1,7 ± 0,1 | 1,00 ± 0,07 | S.2.3 |
| FCS, Liposome model, Genetic algorithm | 3,73 ± 0,20 | 58,0 ± 3,4 | 21,4 ± 0,8 | 1,5 ± 0,1 | 2,02 ± 0,10 | S.2.4 |

| 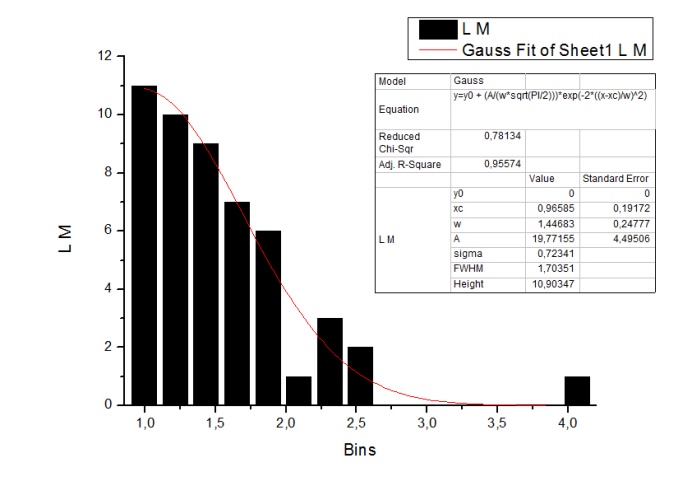 | 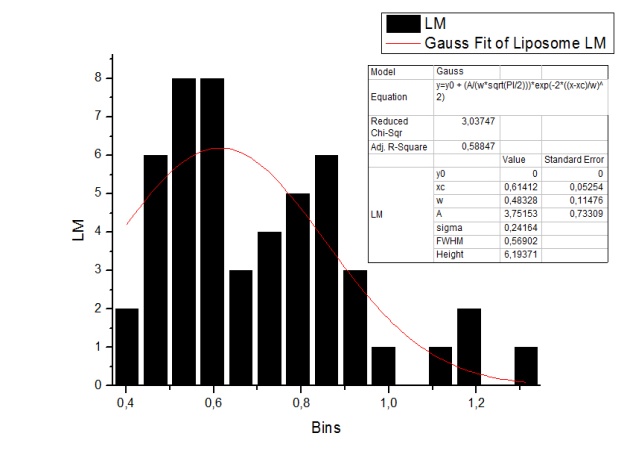 | 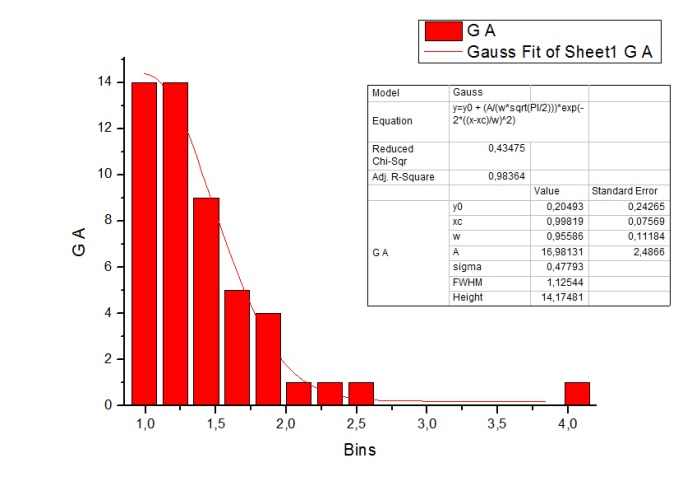 | 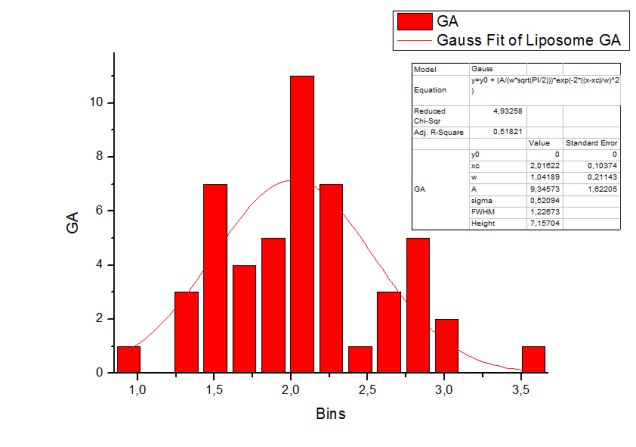 |
| --- | --- | --- | --- |
| Figure S.2.1 | Figure S.2.2 | Figure S.2.3 | Figure S.2.4 |

Atto488-PE; D=100nm; ratio 1:1000

| Technique, model, fitting algorithm | Diffusion [um/s^2^] | Radius of vesicle [nm] | Vesicles spread [nm] | Liposome Concentration [nM] | Focal Volume [fl] | Corresponding figure number |
| --- | --- | --- | --- | --- | --- | --- |
| DLS | 3,97 ± 0,02 | 60,3 ± 0,5 | 21,3 ± 0,5 | - | - | - |
| FCS, Brownian model, LM algorithm | 4,06 ± 0,22 | - | - | 3,70 ± 0,12 | 0,70 ± 0,01 | S.2.5 |
| FCS, Liposome model, LM algorithm | 3,97 ± 0,32 | 60,2 ± 4,4 | 24 ± 14 | 7,61 ± 0,57 | 1,1 ± 0,2 | S.2.6 |
| FCS, Brownian model, Genetic algorithm | 4,00 ± 0,11 | - | - | 3,01 | 0,86 ± 0,16 | S.2.7 |
| FCS, Liposome model, Genetic algorithm | 3,95 ± 0,08 | 54,6 ± 1,3 | 21 ± 1 | 2,22 ± 0,17 | 2,6 ± 0,2 | S.2.8 |

| 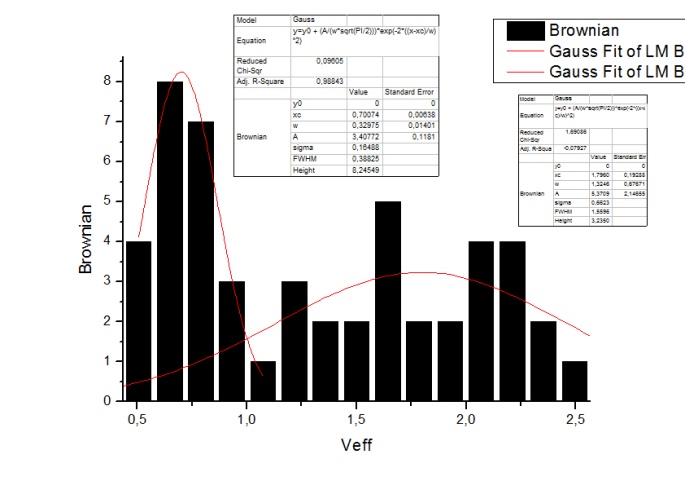 | 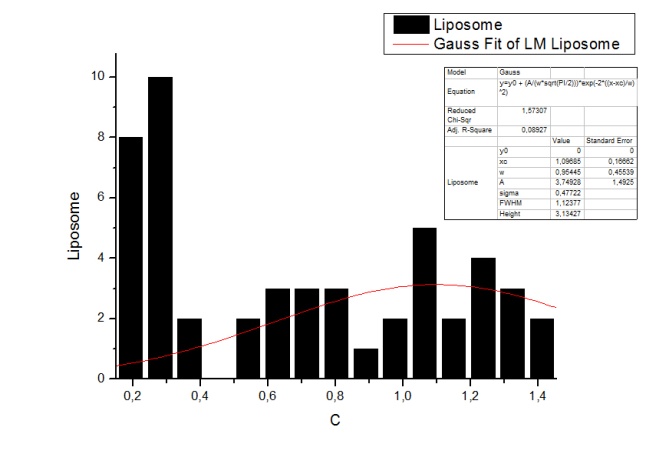 | 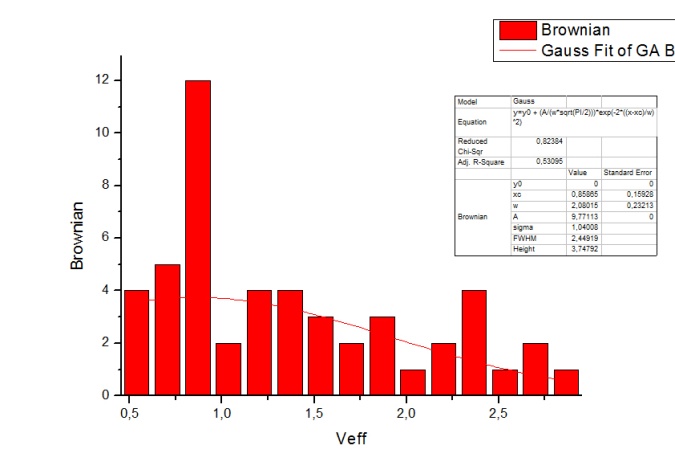 | 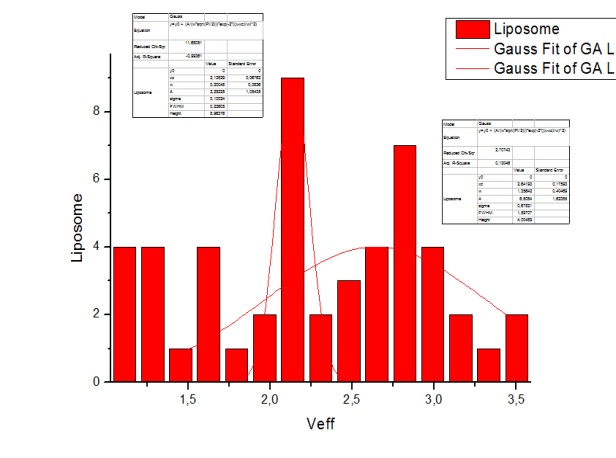 |
| --- | --- | --- | --- |
| Figure S.2.5 | Figure S.2.6 | Figure S.2.7 | Figure S.2.8 |

Atto488-PE; D=50nm; ratio 1:500

| Technique, model, fitting algorithm | Diffusion [um/s^2^] | Radius of vesicle [nm] | Vesicles spread [nm] | Liposome Concentration [nM] | Focal Volume [fl] | Corresponding figure number |
| --- | --- | --- | --- | --- | --- | --- |
| DLS | 6,51 ± 0,03 | 36,8 ± 0,2 | 13,4 ± 1,7 | - | - | - |
| FCS, Brownian model, LM algorithm | 6,60 ± 0,21 | - | - | 4,24 ± 0,06 | 0,87 ± 0,01 | S.2.9 |
| FCS, Liposome model, LM algorithm | 6,27 ± 0,59 | 38,2 ± 3,1 | 9 ± 7 | 16,6 ± 0,3 | 0,36 ± 0,02 | S.2.10 |
| FCS, Brownian model, Genetic algorithm | 6,62 ± 0,19 | - | - | 6,03 ± 0,08 | 0,62 ± 0,01 | S.2.11 |
| FCS, Liposome model, Genetic algorithm | 6,47 ± 0,16 | 33,4 ± 0,9 | 13,1 ± 0,4 | 2,95 ± 0,21 | 2,42 ± 0,21 | S.2.12 |

| 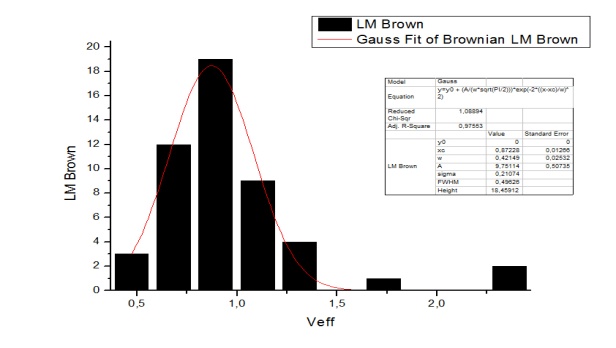 | 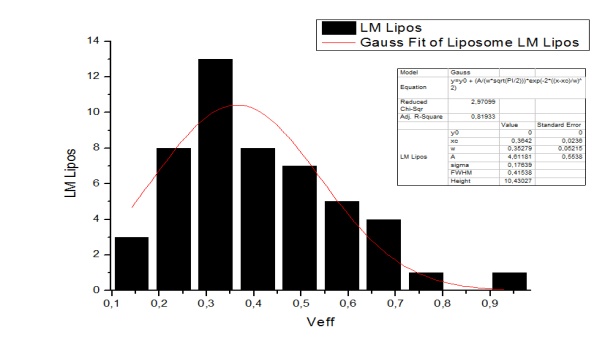 | 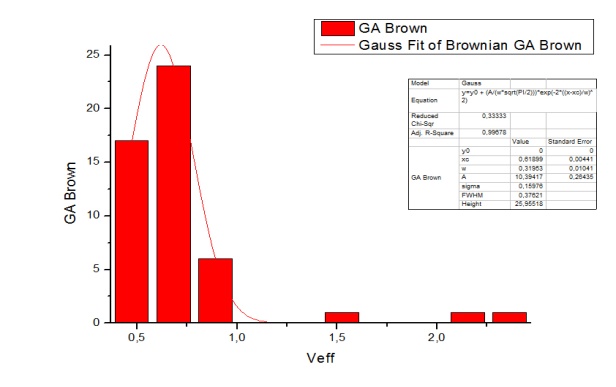 | 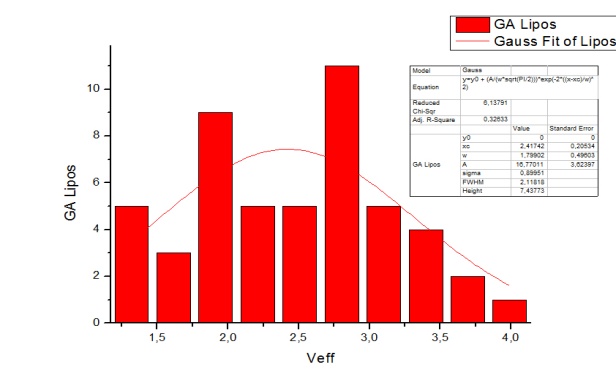 |
| --- | --- | --- | --- |
| Figure S.2.9 | Figure S.2.10 | Figure S.2.11 | Figure S.2.12 |

Atto488-PE; D=50nm; ratio 1:1000

| Technique, model, fitting algorithm | Diffusion [um/s^2^] | Radius of vesicle [nm] | Vesicles spread [nm] | Liposome Concentration [nM] | Focal Volume [fl] | Corresponding figure number |
| --- | --- | --- | --- | --- | --- | --- |
| DLS | 6,22 ± 0,09 | 38,5 ± 0,8 | 15,1 ± 2,1 | - | - | - |
| FCS, Brownian model, LM algorithm | 6,11 ± 0,14 | - | - | 9,8 ± 0,4 | 0,30 ± 0,08 | S.2.13 |
| FCS, Liposome model, LM algorithm | 4,88 ± 0,50 | 48 ± 4 | 5 ± 3 | 25 ± 3 | 0,09 ± 0,03 | S.2.14 |
| FCS, Brownian model, Genetic algorithm | 6,29 ± 0,22 | - | - | 6,8 ± 0,4 | 0,43 ± 0,07 | S.2.15 |
| FCS, Liposome model, Genetic algorithm | 6,15 ± 0,22 | 35,1 ± 1,4 | 14,7 ± 0,4 | 3,0 ± 0,3 | 2,17 ± 0,18 | S.2.16 |

| 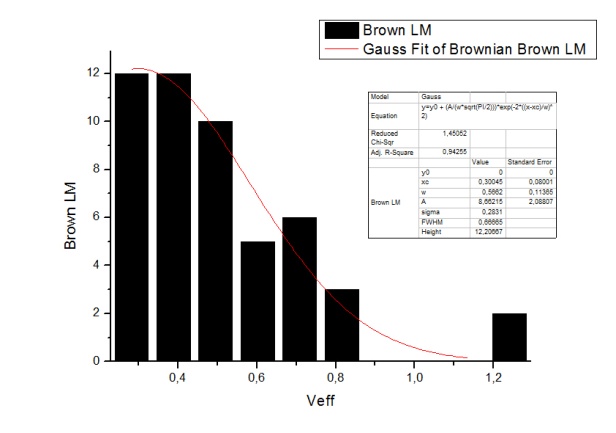 | 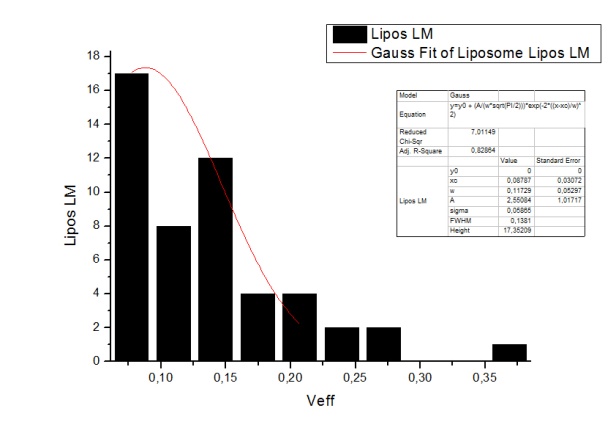 | 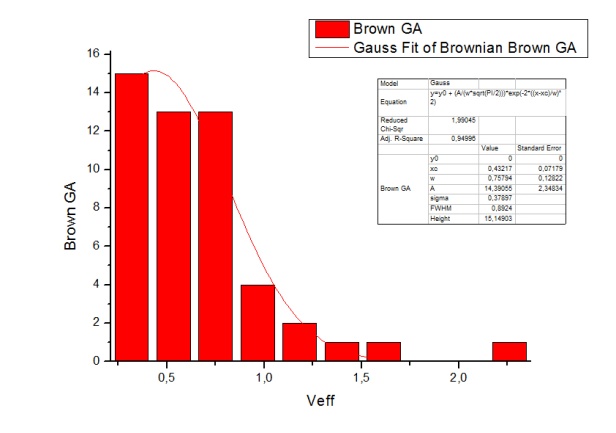 | 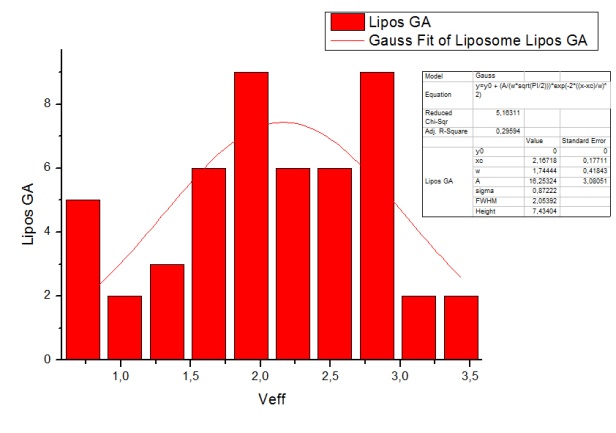 |
| --- | --- | --- | --- |
| Figure S.2.13 | Figure S.2.14 | Figure S.2.15 | Figure S.2.16 |

βBodipy FL, D=100nm, ratio 1:500

| Technique, model, fitting algorithm | Diffusion [um/s^2^] | Radius of vesicle [nm] | Vesicles spread [nm] | Liposome Concentration [nM] | Focal Volume [fl] | Corresponding figure number |
| --- | --- | --- | --- | --- | --- | --- |
| DLS | 4,23 | 55,63 ± 0,65 | 21,3 ± 1,2 | - | - | - |
| FCS, Brownian model, LM algorithm | 4,48 ± 0,21 | - | - | 1,57 ± 0,04 | 1,99 ± 0,09 | S.3.1 |
| FCS, Liposome model, LM algorithm | 4,59 ± 0,18 | 52 ± 2 | 33 ± 4 | 5,6 ± 0,5 | 1,05 ± 0,03 | S.3.2 |
| FCS, Brownian model, Genetic algorithm | 4,24 ± 0,13 | - | - | 1,1 ± 0,3 | 1,71 ± 0,07 | S.3.3 |
| FCS, Liposome model, Genetic algorithm | 4,18 ± 0,13 | 52 ± 2 | 20,3 ± 0,8 | 2,3 ± 0,2 | 2,53 ± 0,13 | S.3.4 |

| 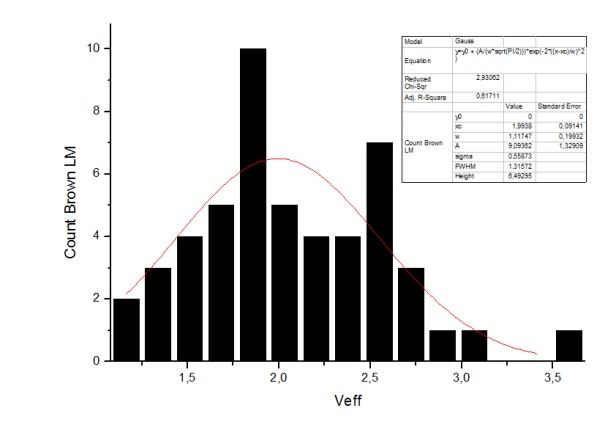 | 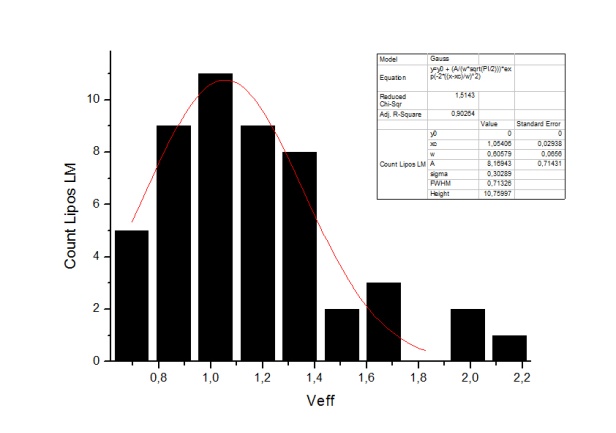 | 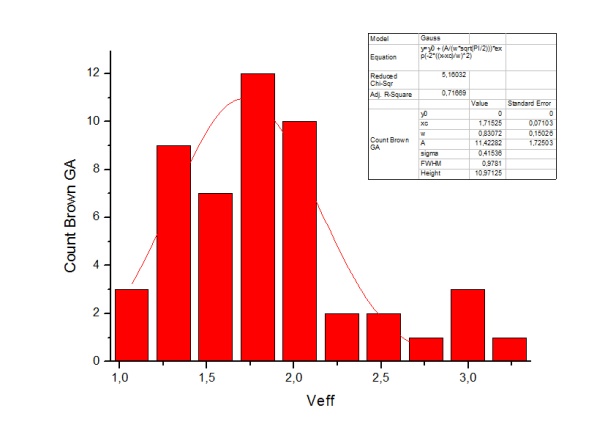 | 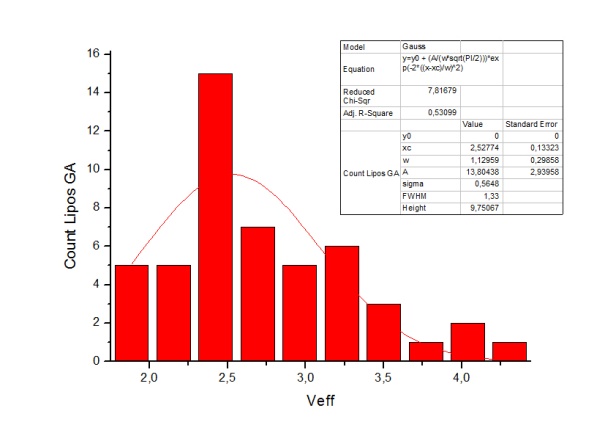 |
| --- | --- | --- | --- |
| Figure S.3.1 | Figure S.3.2 | Figure S.3.3 | Figure S.3.4 |

βBodipy FL, D=100nm, ratio 1:1000

| Technique, model, fitting algorithm | Diffusion [um/s^2^] | Radius of vesicle [nm] | Vesicles spread [nm] | Liposome Concentration [nM] | Focal Volume [fl] | Corresponding figure number |
| --- | --- | --- | --- | --- | --- | --- |
| DLS | 4,09 | 58,47 ± 0,45 | 18,9 ± 2,3 | - | - | - |
| FCS, Brownian model, LM algorithm | 4,27 ± 0,21 | - | - | 2,36 ± 0,11 | 1,48 ± 0,03 | S.3.5 |
| FCS, Liposome model, LM algorithm | 4,12 ± 0,29 | 58 ± 4 | 23 ± 12 | 9,0 ± 0,4 | 0,73 ± 0,03 | S.3.6 |
| FCS, Brownian model, Genetic algorithm | 4,10 ± 0,13 | - | - | 2,40 ± 0,09 | 1,43 ± 0,03 | S.3.7 |
| FCS, Liposome model, Genetic algorithm | 4,07 ± 0,09 | 52,9 ± 1,3 | 18,46 ± 0,51 | 2,19 ± 0,11 | 2,49 ± 0,14 | S.3.8 |

| 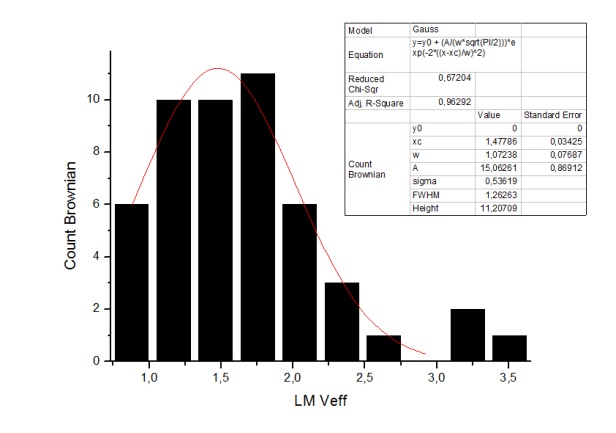 | 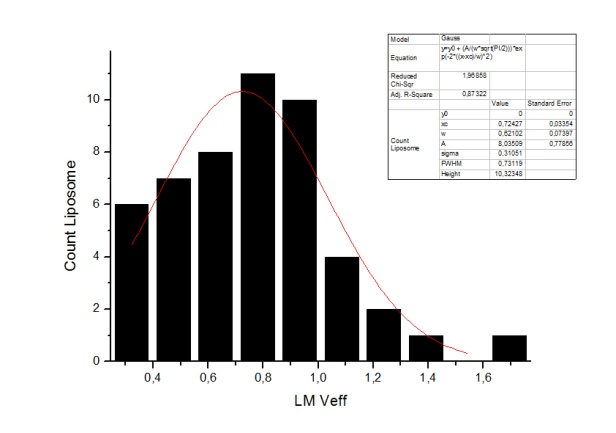 | 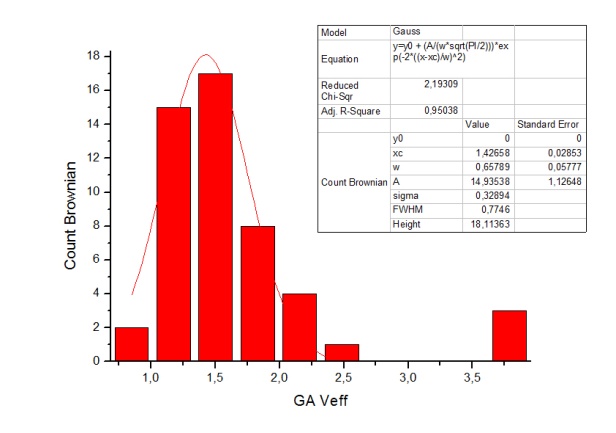 | 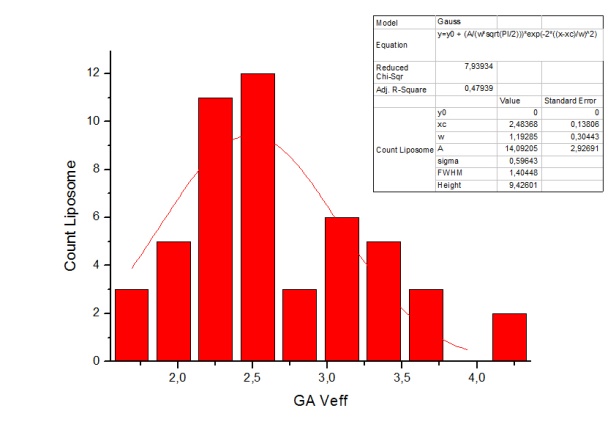 |
| --- | --- | --- | --- |
| Figure S.3.5 | Figure S.3.6 | Figure S.3.7 | Figure S.3.8 |

βBodipy FL, D=50nm, ratio 1:500

| Technique, model, fitting algorithm | Diffusion [um/s^2^] | Radius of vesicle [nm] | Vesicles spread [nm] | Liposome Concentration [nM] | Focal Volume [fl] | Corresponding figure number |
| --- | --- | --- | --- | --- | --- | --- |
| DLS | 7,08 | 33,8 ± 0,6 | 14,9 ± 1,6 | - | - | - |
| FCS, Brownian model, LM algorithm | 7,3 ± 0,3 | - | - | 5,4 ± 0,3 | 1,26 ± 0,02 | S.3.9 |
| FCS, Liposome model, LM algorithm | 6,86 ± 0,83 | 35 ± 4 | 12 ± 7 | 51,9 ± 0,7 | 0,46 ± 0,18 | S.3.10 |
| FCS, Brownian model, Genetic algorithm | 7,03 ± 0,22 | - | - | 9,68 ± 0,05 | 1,306 ± 0,001 | S.3.11 |
| FCS, Liposome model, Genetic algorithm | 7,05 ± 0,18 | 31 ± 1 | 14,5 ± 0,4 | 6,8 ± 0,3 | 4,08 ± 0,22 | S.3.12 |

| 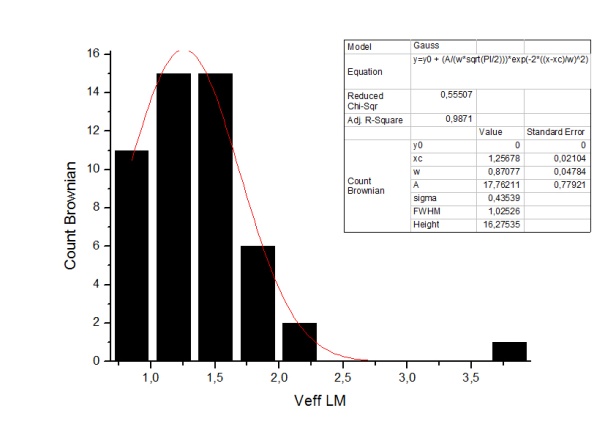 | 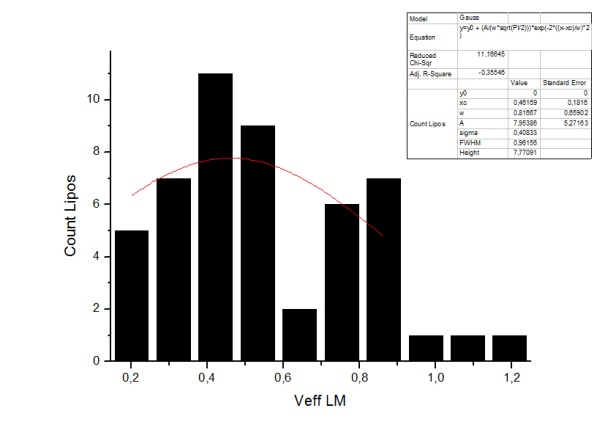 | 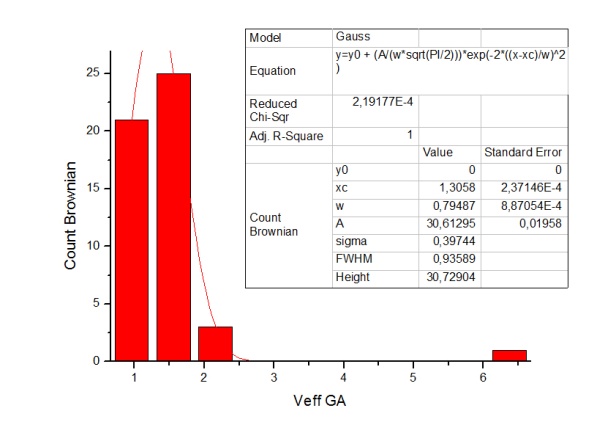 | 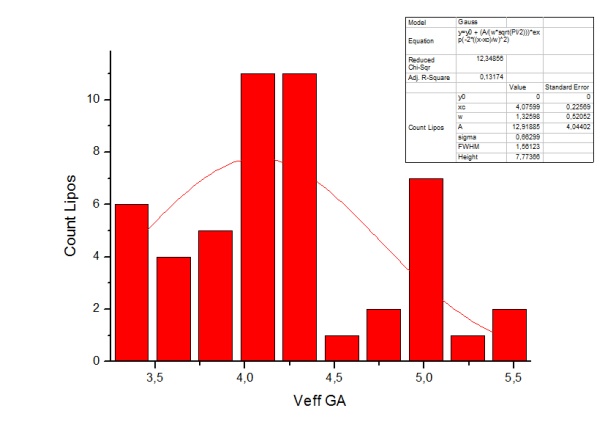 |
| --- | --- | --- | --- |
| Figure S.3.9 | Figure S.3.10 | Figure S.3.11 | Figure S.3.12 |

βBodipy FL, D=50nm, ratio 1:1000

| Technique, model, fitting algorithm | Diffusion [um/s^2^] | Radius of vesicle [nm] | Vesicles spread [nm] | Liposome Concentration [nM] | Focal Volume [fl] | Corresponding figure number |
| --- | --- | --- | --- | --- | --- | --- |
| DLS | 6,47 | 37,0 ± 2,5 | 19,16 ± 2,4 | - | - | - |
| FCS, Brownian model, LM algorithm | 6,49 ± 0,17 | - | - | 12,6 ± 0,3 | 1,00 ± 0,03 | S.3.13 |
| FCS, Liposome model, LM algorithm | 5,48 ± 0,81 | 42 ± 6 | 8 ± 5 | 340±32 | 1,000 ± 0,001 | S.3.14 |
| FCS, Brownian model, Genetic algorithm | 6,43 ± 0,23 | - | - | 218 ± 68 | 0,10 ± 0,04 | S.3.15 |
| FCS, Liposome model, Genetic algorithm | 6,41 ± 0,24 | 34 ± 2 | 18,7 ± 0,5 | 6,76 ± 0,14 | 2,34 ± 0,04 | S.3.16 |

| 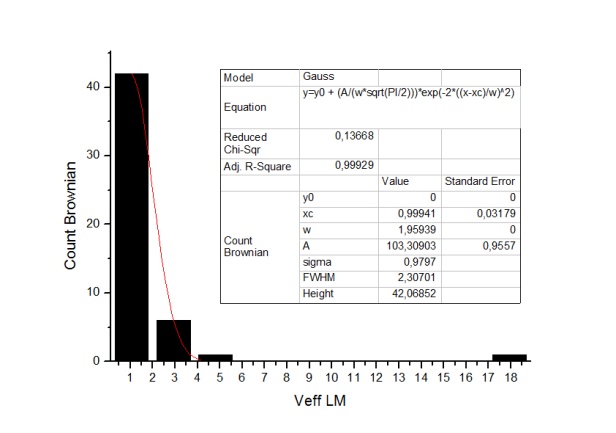 | 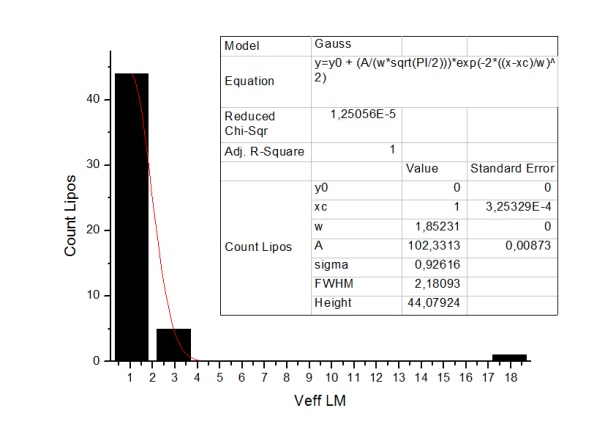 | 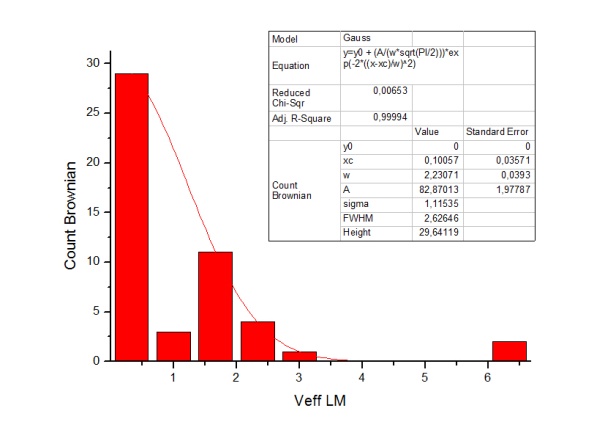 | 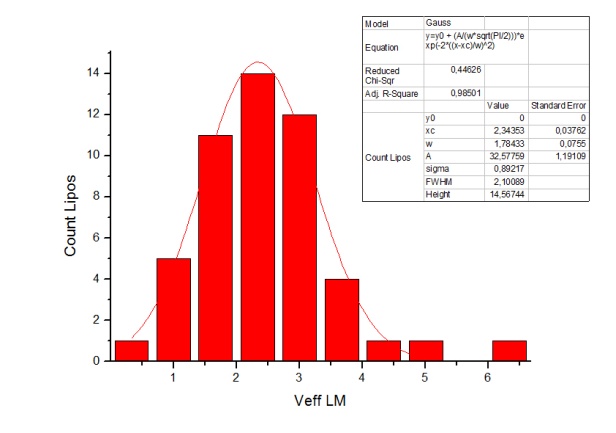 |
| --- | --- | --- | --- |
| Figure S.3.13 | Figure S.3.14 | Figure S.3.15 | Figure S.3.16 |

Fluoresceince-PE, D=100nm, ratio 1:500

| Technique, model, fitting algorithm | Diffusion [um/s^2^] | Radius of vesicle [nm] | Vesicles spread [nm] | Liposome Concentration [nM] | Focal Volume [fl] | Corresponding figure number |
| --- | --- | --- | --- | --- | --- | --- |
| DLS | 4,39 ± 0,04 | 54,56 ± 0,65 | 17,9 ± 1,2 | - | - | - |
| FCS, Brownian model, LM algorithm | 4,52 ± 0,17 | - | - | 4,1 ± 0,3 | 2,02 ± 0,09 | S.4.1 |
| FCS, Liposome model, LM algorithm | 4,4 ± 0,5 | 55,1 +- 6,3 | 19 +- 13 | 15,3 ± 4,4 | 0,67 ± 0,11 | S.4.2 |
| FCS, Brownian model, Genetic algorithm | 4,39 ± 0,13 | - | - | 6,09 ± 0,12 | 1,43 ± 0,15 | S.4.3 |
| FCS, Liposome model, Genetic algorithm | 4,32 ± 0,15 | 50 +- 2 | 17,3 +- 0,6 | 2,40 ± 0,05 | 2,40 ± 0,04 | S.4.4 |

| 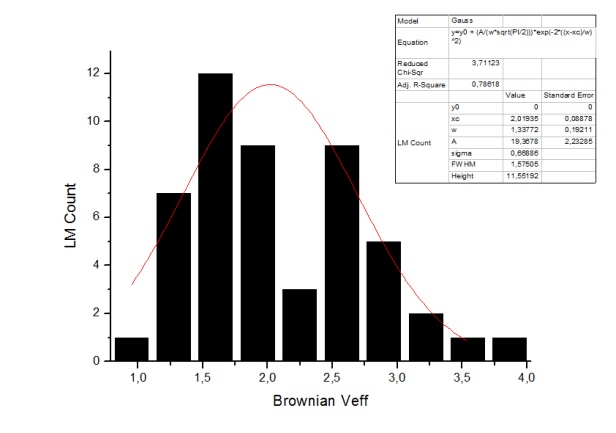 | 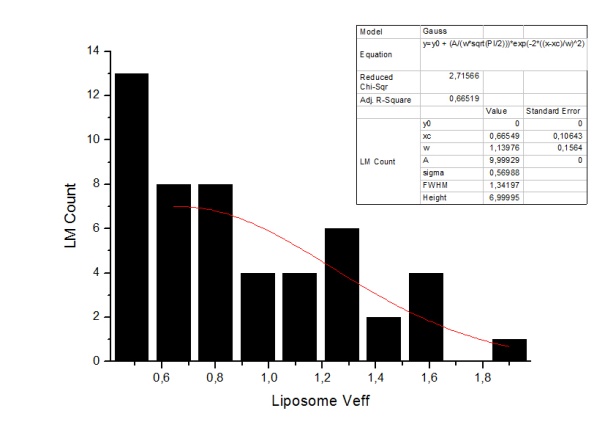 | 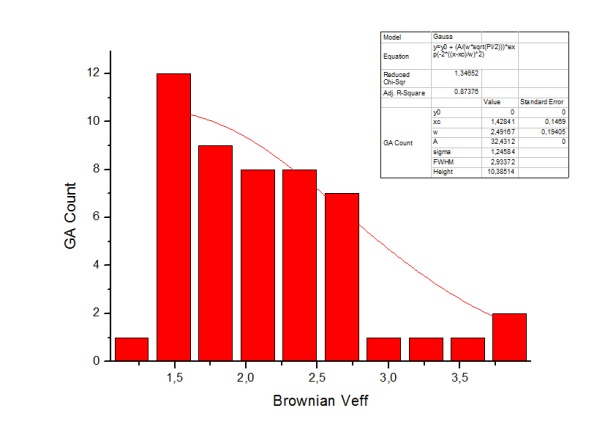 | 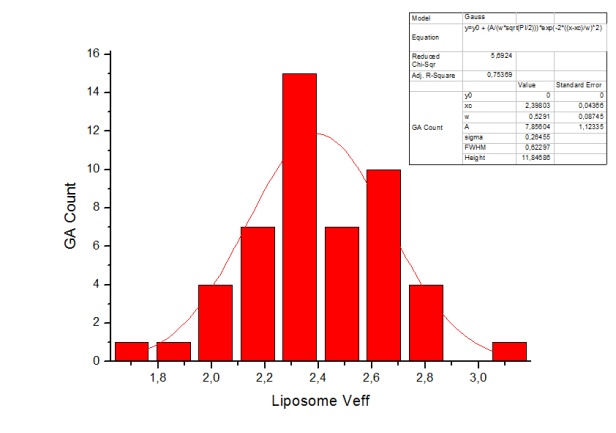 |
| --- | --- | --- | --- |
| Figure S.4.1 | Figure S.4.2 | Figure S.4.3 | Figure S.4.4 |

Fluoresceince-PE, D=100nm, ratio 1:1000

| Technique, model, fitting algorithm | Diffusion [um/s^2^] | Radius of vesicle [nm] | Vesicles spread [nm] | Liposome Concentration [nM] | Focal Volume [fl] | Corresponding figure number |
| --- | --- | --- | --- | --- | --- | --- |
| DLS | 4,34 ± 0,01 | 55,15 ± 0,05 | 19,2 ± 1,2 | - | - | - |
| FCS, Brownian model, LM algorithm | 4,31 ± 0,06 | - | - | 5,88 ± 0,13 | 0,898 ± 0,051 | S.4.5 |
| FCS, Liposome model, LM algorithm | 4,34 ± 0,15 | 62,8 ± 3,3 | 6,2 ± 3,1 | 30,0 ± 0,7 | 0,26 ± 0,03 | S.4.6 |
| FCS, Brownian model, Genetic algorithm | 3,75 ± 0,24 | - | - | 4,5 ± 1,0 | 1,084 ± 0,024 | S.4.7 |
| FCS, Liposome model, Genetic algorithm | 4,34 ± 0,02 | 49,7 ± 0,2 | 18,8 ± 0,5 | 33 ± 2 | 2,034 ± 0,044 | S.4.8 |

| 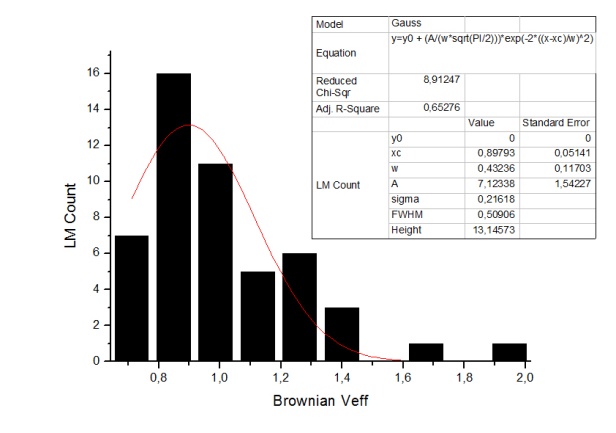 | 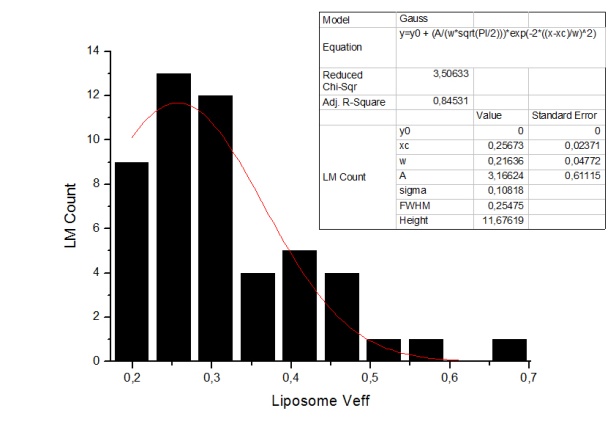 | 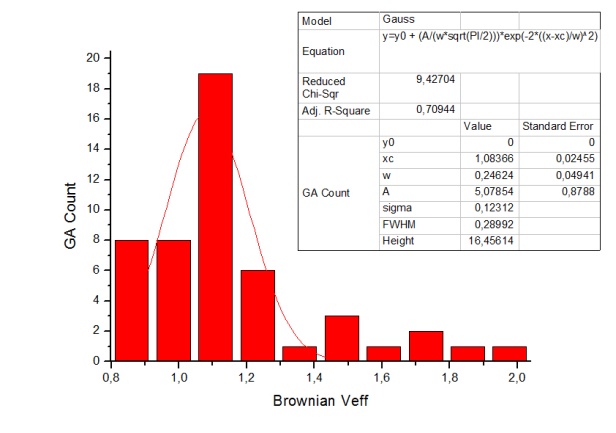 | 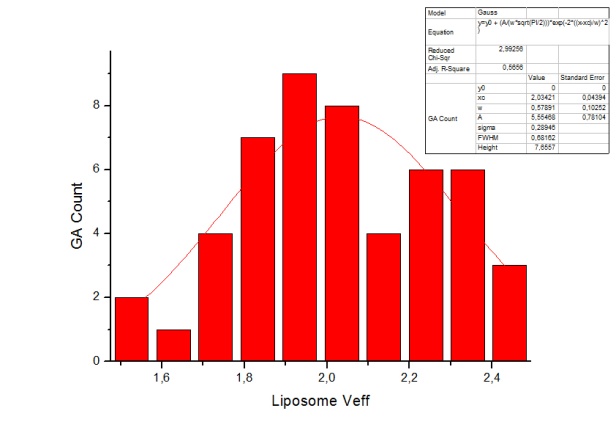 |
| --- | --- | --- | --- |
| Figure S.4.5 | Figure S.4.6 | Figure S.4.7 | Figure S.4.8 |

Fluoresceince-PE, D=50nm, ratio 1:500

| Technique, model, fitting algorithm | Diffusion [um/s^2^] | Radius of vesicle [nm] | Vesicles spread [nm] | Liposome Concentration [nM] | Focal Volume [fl] | Corresponding figure number |
| --- | --- | --- | --- | --- | --- | --- |
| DLS | 7,20 ± 0,07 | 33,30 ± 0,42 | 12,6 ± 1,2 | - | - | - |
| FCS, Brownian model, LM algorithm | 7,4 ± 0,3 | - | - | 4,8 ± 0,1 | 2,35 ± 0,03 | S.4.9 |
| FCS, Liposome model, LM algorithm | 7 ± 1 | 33 ± 4 | 12,7 ± 7,1 | 19,6 ± 0,8 | 1,00 ± 0,04 | S.4.10 |
| FCS, Brownian model, Genetic algorithm | 7,15 ± 0,21 | - | - | 8,6 ± 1,3 | 2,01 ± 0,03 | S.4.11 |
| FCS, Liposome model, Genetic algorithm | 7,0 ± 0,4 | 31 ± 2 | 12,2 ± 0,4 | 10,4 ± 1,5 | 2,12 ± 0,09 | S.4.12 |

| 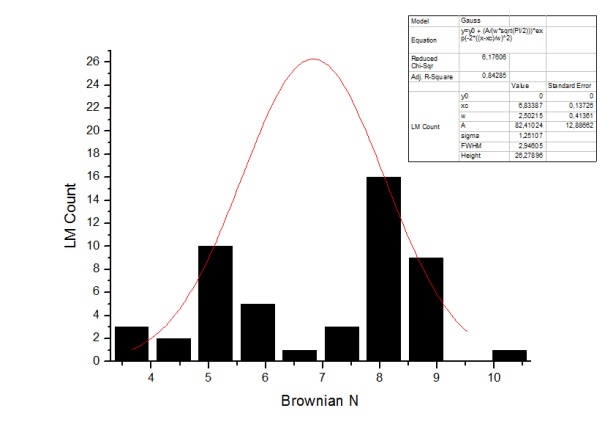 | 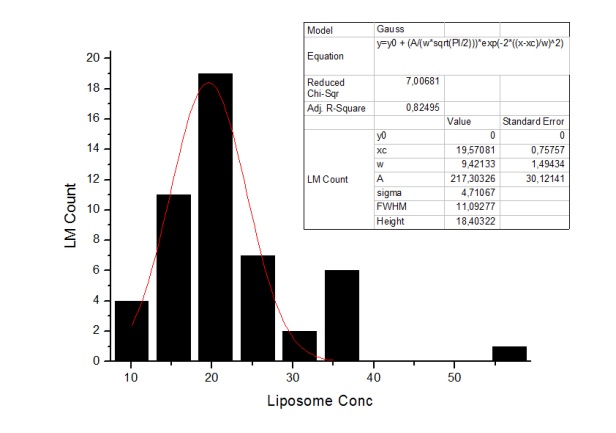 | 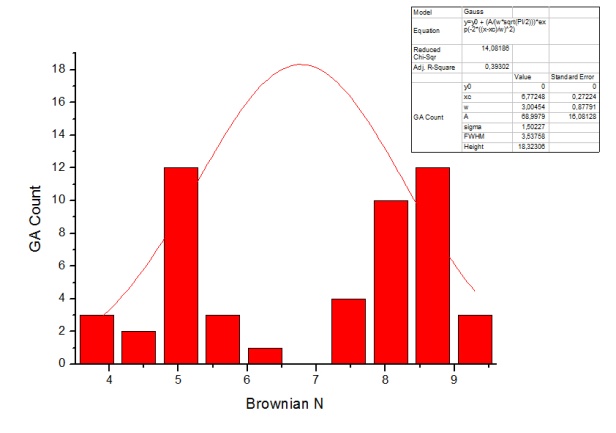 | 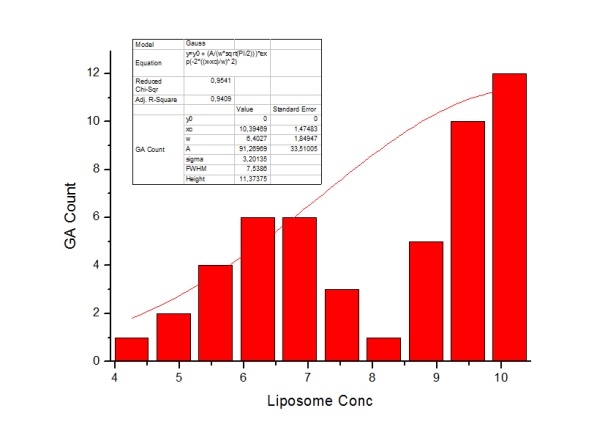 |
| --- | --- | --- | --- |
| Figure S.4.9 | Figure S.4.10 | Figure S.4.11 | Figure S.4.12 |

Fluoresceince-PE, D=50nm, ratio 1:1000

| Technique, model, fitting algorithm | Diffusion [um/s^2^] | Radius of vesicle [nm] | Vesicles spread [nm] | Liposome Concentration [nM] | Focal Volume [fl] | Corresponding figure number |
| --- | --- | --- | --- | --- | --- | --- |
| DLS | 6,15 ± 0,23 | 38,9 ± 2,1 | 17,4 ± 0,7 | - | - | - |
| FCS, Brownian model, LM algorithm | 6,2 ± 0,2 | - | - | 5,5 ± 0,08 | 1,76 ± 0,06 | S.4.13 |
| FCS, Liposome model, LM algorithm | 5,4 ± 0,9 | 44,0 ± 6,3 | 9 ± 6 | 17 ± 9 | 0,55 ± 0,05 | S.4.14 |
| FCS, Brownian model, Genetic algorithm | 6,12 ± 0,21 | - | - | 5,33 ± 0,17 | 1,9 ± 0,2 | S.4.15 |
| FCS, Liposome model, Genetic algorithm | 6,145 ± 0,006 | 35,08 ± 0,04 | 16,9 ± 0,5 | 7,89 ± 0,08 | 2,41 ± 0,05 | S.4.16 |

| 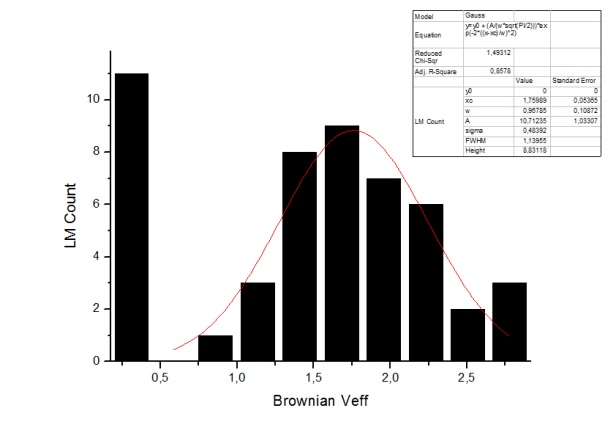 | 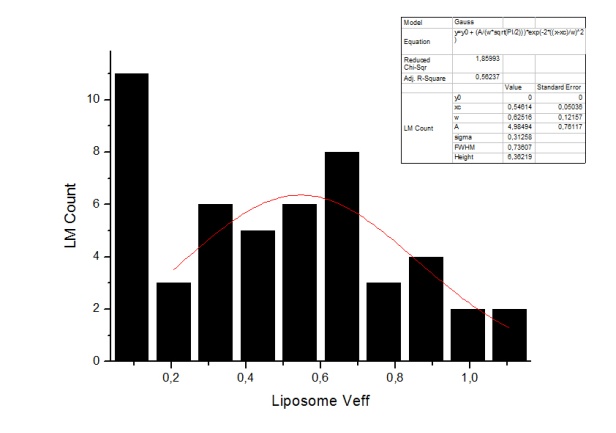 | 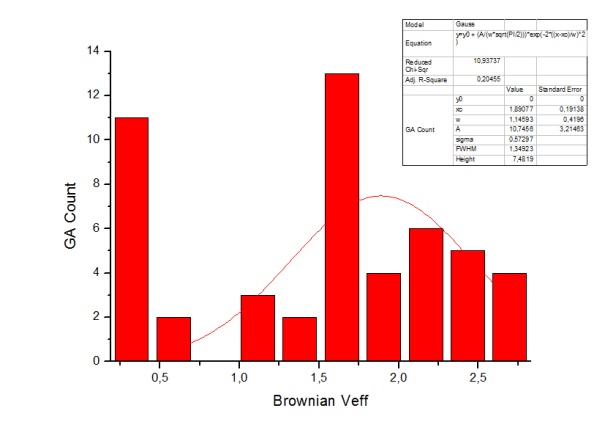 | 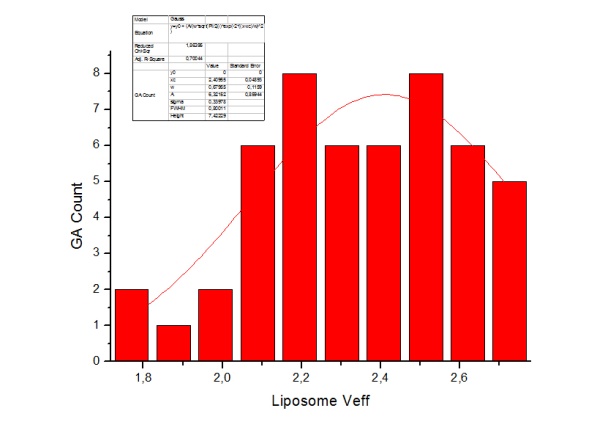 |
| --- | --- | --- | --- |
| Figure S.4.13 | Figure S.4.14 | Figure S.4.15 | Figure S.4.16 |

NBD-PE, D=100nm, ratio 1:500

| Technique, model, fitting algorithm | Diffusion [um/s^2^] | Radius of vesicle [nm] | Vesicles spread [nm] | Liposome Concentration [nM] | Focal Volume [fl] | Corresponding figure number |
| --- | --- | --- | --- | --- | --- | --- |
| DLS | 4,01 ± 0,04 | 59,71 ± 0,93 | 24,0 ± 2,2 | - | - | - |
| FCS, Brownian model, LM algorithm | 4,08 ± 0,13 | - | - | 3,5 ± 0,08 | 1,57 ± 0,17 | S.5.1 |
| FCS, Liposome model, LM algorithm | 3,8 ± 0,5 | 63,2 ± 7,2 | 16 ± 12 | 0,4 ± 6,4 | 0,47 ± 0,05 | S.5.2 |
| FCS, Brownian model, Genetic algorithm | 4,02 ± 0,11 | - | - | 3,27 ± 0,09 | 1,80 ± 0,06 | S.5.3 |
| FCS, Liposome model, Genetic algorithm | 3,87 ± 0,23 | 56 ± 4 | 23,0 ± 0,7 | 4,00 ± 0,08 | 2,19 ± 0,03 | S.5.4 |

| 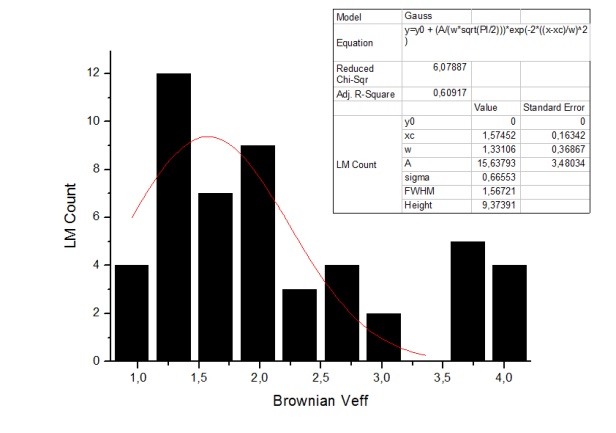 | 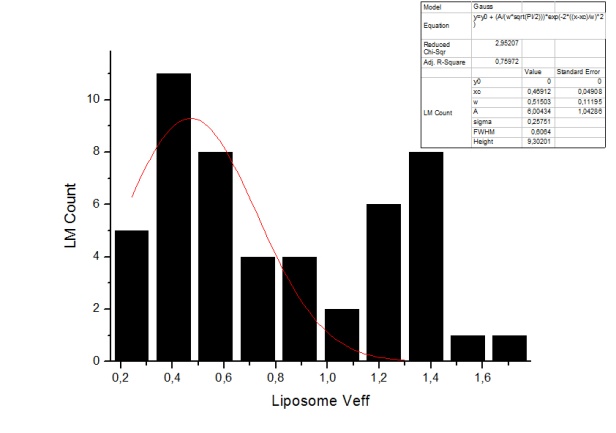 | 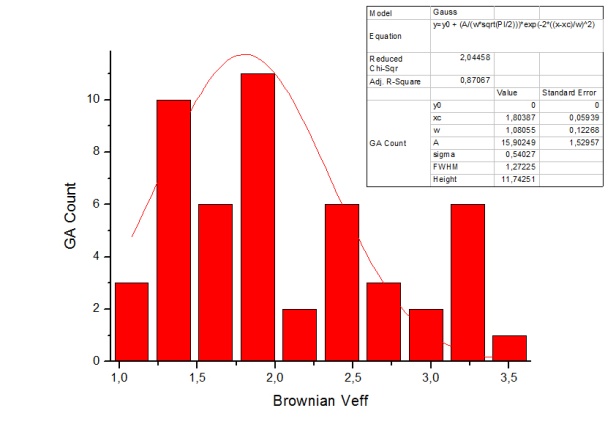 | 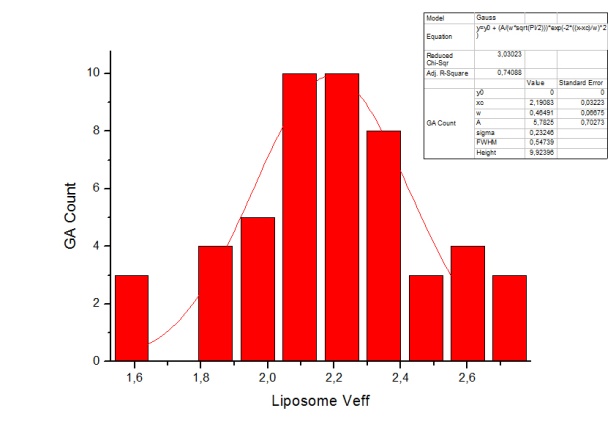 |
| --- | --- | --- | --- |
| Figure S.5.1 | Figure S.5.2 | Figure S.5.3 | Figure S.5.4 |

NBD-PE, D=100nm, ratio 1:1000

| Technique, model, fitting algorithm | Diffusion [um/s^2^] | Radius of vesicle [nm] | Vesicles spread [nm] | Liposome Concentration [nM] | Focal Volume [fl] | Corresponding figure number |
| --- | --- | --- | --- | --- | --- | --- |
| DLS | 4,15 ± 0,04 | 57,8 ± 0,7 | 17,5 ± 1,3 | - | - | - |
| FCS, Brownian model, LM algorithm | 4,18 ± 0,11 | - | - | 3,66 ± 0,08 | 1,67 ± 0,09 | S.5.5 |
| FCS, Liposome model, LM algorithm | 3,7 ± 0,6 | 65 ± 10 | 10 ± 11 | 16 ± 1 | 0,51 ± 0,06 | S.5.6 |
| FCS, Brownian model, Genetic algorithm | 4,21 ± 0,13 | - | - | 3,3 ± 0,2 | 1,75 ± 0,12 | S.5.7 |
| FCS, Liposome model, Genetic algorithm | 4,07 ± 0,19 | 53 ± 3 | 17,20 ± 0,43 | 5,0 ± 0,7 | 2,08 ± 0,04 | S.5.8 |

| 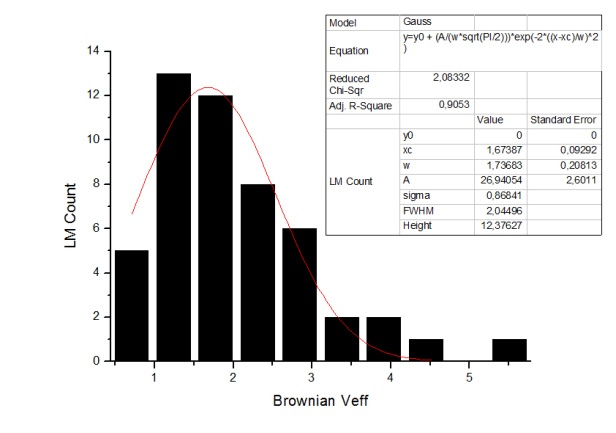 | 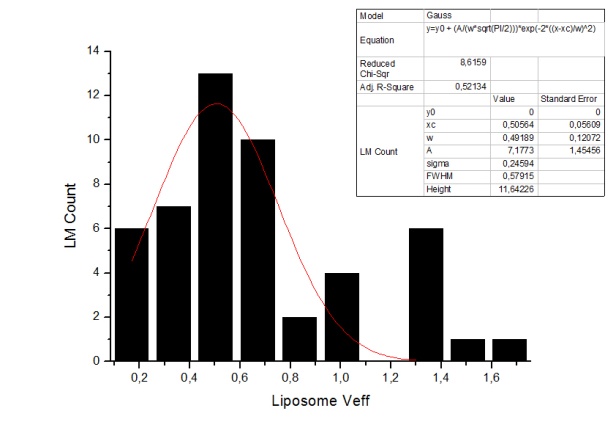 | 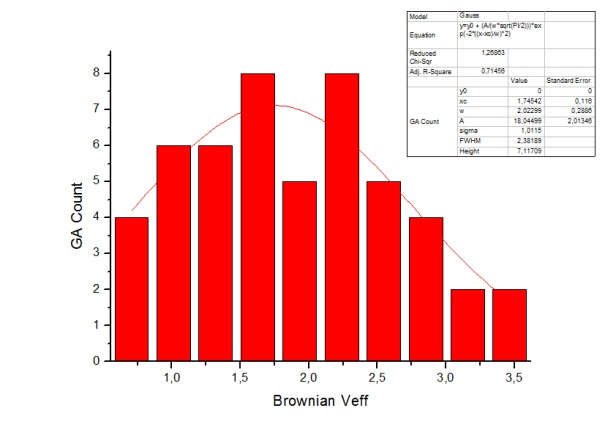 | 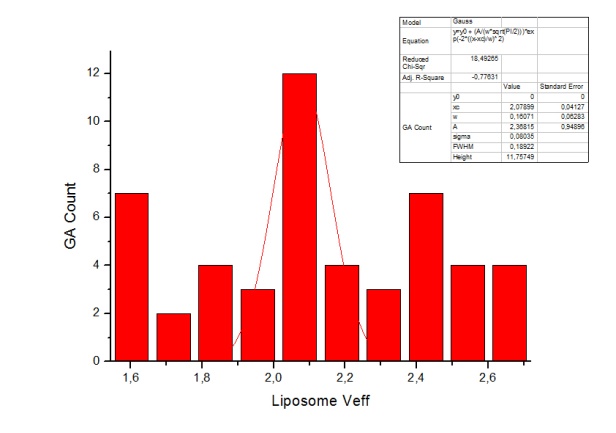 |
| --- | --- | --- | --- |
| Figure S.5.5 | Figure S.5.6 | Figure S.5.7 | Figure S.5.8 |

NBD-PE, D=50nm, ratio 1:500

| Technique, model, fitting algorithm | Diffusion [um/s^2^] | Radius of vesicle [nm] | Vesicles spread [nm] | Liposome Concentration [nM] | Focal Volume [fl] | Corresponding figure number |
| --- | --- | --- | --- | --- | --- | --- |
| DLS | 6,68 ± 0,02 | 35,75 ± 0,13 | 9,6 ± 0,8 | - | - | - |
| FCS, Brownian model, LM algorithm | 6,69 ± 0,12 | - | - | 3,64 ± 0,02 | 2,42 ± 0,17 | S.5.9 |
| FCS, Liposome model, LM algorithm | 6,2 ± 1,3 | 40 ± 6 | 5 ± 6 | 6,11 ± 0,22 | 0,56 ± 0,16 | S.5.10 |
| FCS, Brownian model, Genetic algorithm | 6,69 ± 0,22 | - | - | 3,68 ± 0,15 | 2,20 ± 0,04 | S.5.11 |
| FCS, Liposome model, Genetic algorithm | 6,53 ± 0,35 | 33 ± 2 | 9,3 ± 0,3 | 5,7 ± 0,2 | 2,41 ± 0,03 | S.5.12 |

| 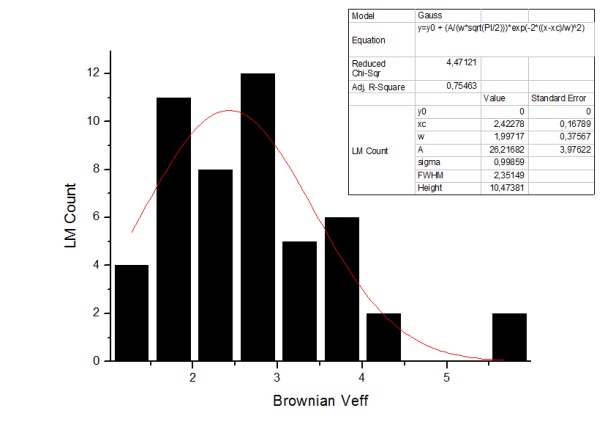 | 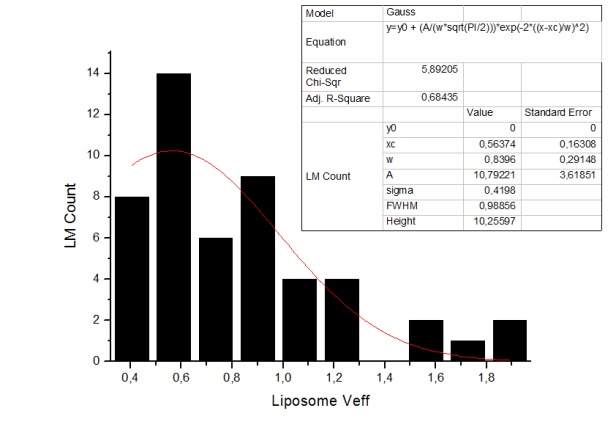 | 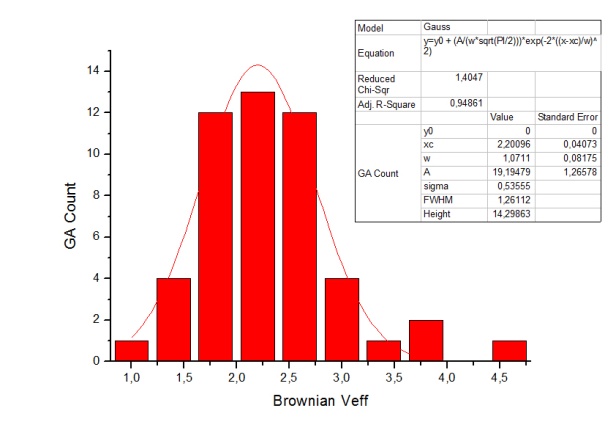 | 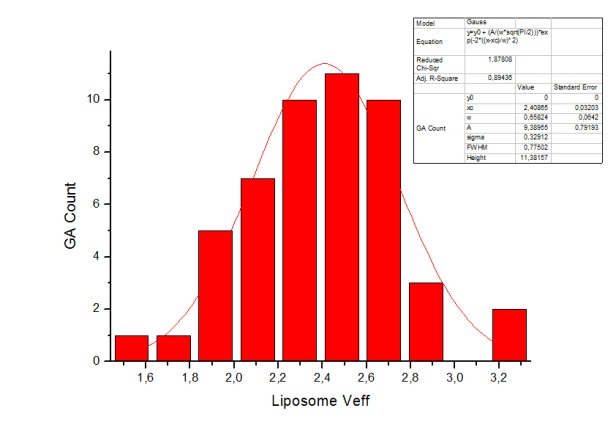 |
| --- | --- | --- | --- |
| Figure S.5.9 | Figure S.5.10 | Figure S.5.11 | Figure S.5.12 |

NBD-PE, D=50nm, ratio 1:1000

| Technique, model, fitting algorithm | Diffusion [um/s^2^] | Radius of vesicle [nm] | Vesicles spread [nm] | Liposome Concentration [nM] | Focal Volume [fl] | Corresponding figure number |
| --- | --- | --- | --- | --- | --- | --- |
| DLS | 6,61 ± 0,03 | 36,3 ± 0,3 | 12,0 ± 1,3 | - | - | - |
| FCS, Brownian model, LM algorithm | 6,63 ± 0,17 | - | - | 16,45 ± 0,54 | 0,52 ± 0,50 | S.5.13 |
| FCS, Liposome model, LM algorithm | 6 ± 1 | 43 ± 7 | 6 ± 7 | 71 ± 5 | 0,165 ± 0,012 | S.5.14 |
| FCS, Brownian model, Genetic algorithm | 6,66 ± 0,24 | - | - | 12,2 ± 0,3 | 0,79 ± 0,05 | S.5.15 |
| FCS, Liposome model, Genetic algorithm | 6,51 ± 0,31 | 33 ± 2 | 12,7 ± 0,3 | 6,63 ± 0,34 | 2,22 ± 0,04 | S.5.16 |

| 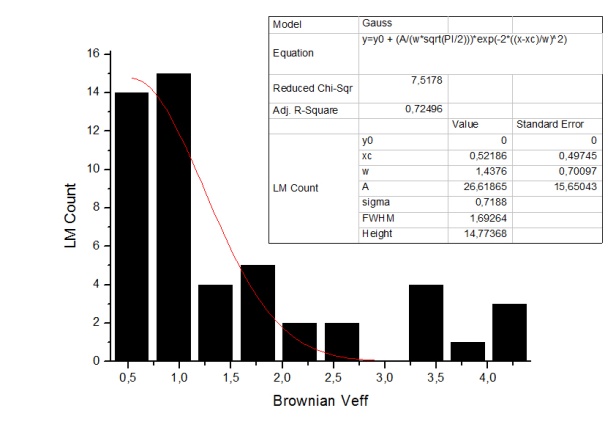 | 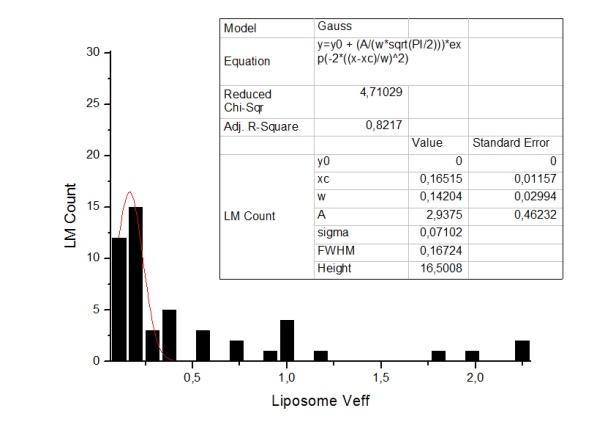 | 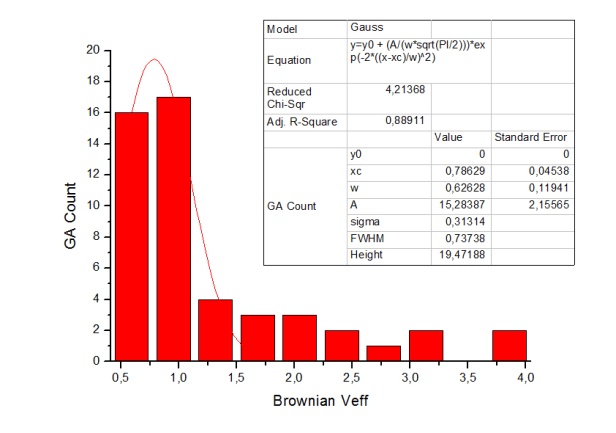 | 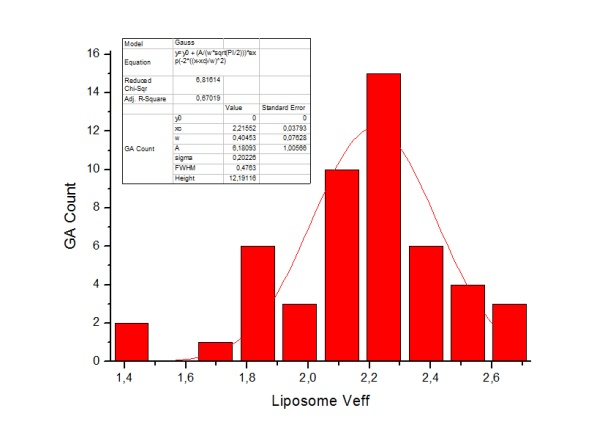 |
| --- | --- | --- | --- |
| Figure S.5.13 | Figure S.5.14 | Figure S.5.15 | Figure S.5.16 |

# Results of DLS measurements.

Each sample was measured 3 times at attenuator set to value of 8 or 9 depending on the sample. All samples were measured at 24 °C. Plots bellow show size distributions for each sample separately. Each line represents individual measurement. Results of size measurements are summarized in the table included in the paper.

Atto488-PE; D=100nm

| 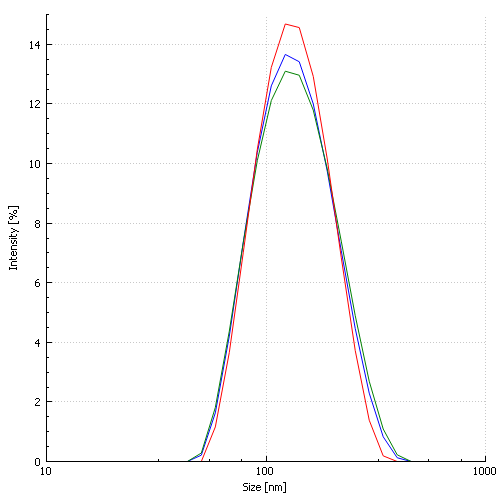 | 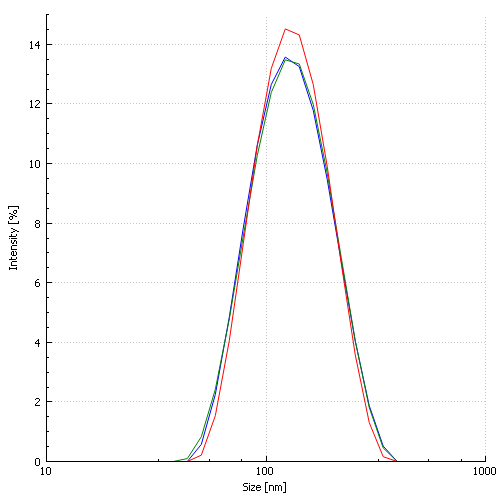 |
| --- | --- |
| Figure S.6.1. Size distributions of Atto488-labelled liposome population with the dye to lipid molar ratio equals to 1:500. | Figure S.6.2. Size distributions of Atto488-labelled liposome population with the dye to lipid molar ratio equals to 1:1000. |

Atto488-PE; D=50nm

| 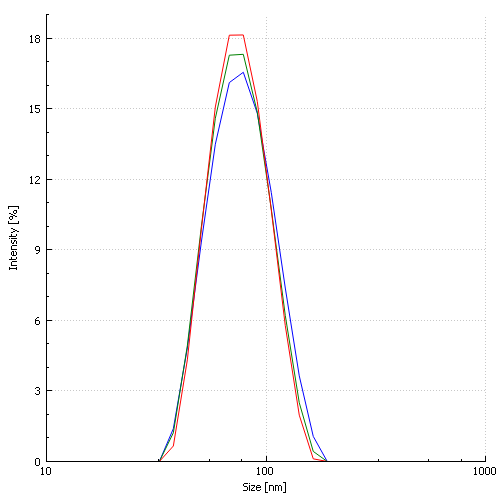 | 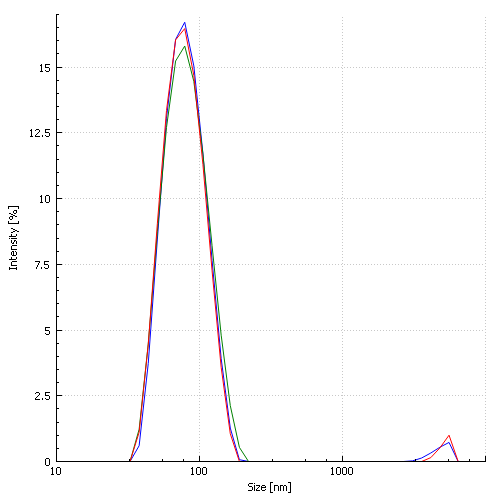 |
| --- | --- |
| Figure S.6.3. Size distributions of Atto488-labelled liposome population with the dye to lipid molar ratio equals to 1:500. | Figure S.6.4. Size distribution of Atto488-labelled liposome population with the dye to lipid molar ratio equals to 1:1000. |

βBodipy FL; D=100nm

| 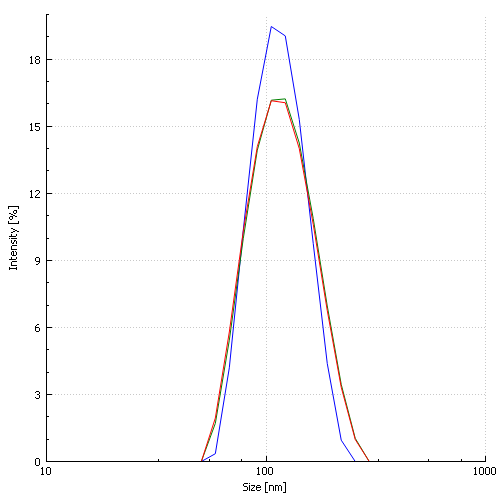 | 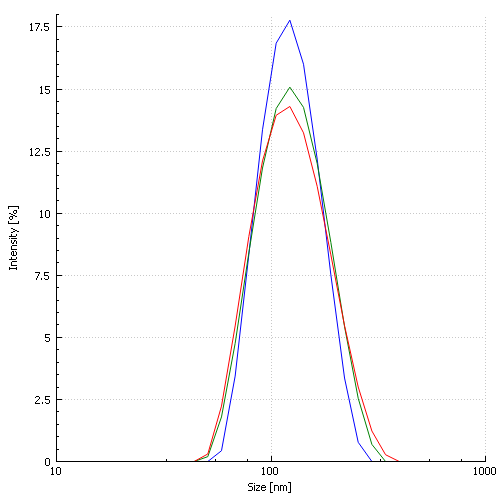 |
| --- | --- |
| Figure S.6.5. Size distribution of βBodipy FL-labelled liposome population with the dye to lipid molar ratio equals to 1:500. | Figure S.6.6. Size distribution of βBodipy FL-labelled liposome population with the dye to lipid molar ratio equals to 1:1000. |

βBodipy FL; D=50nm

| 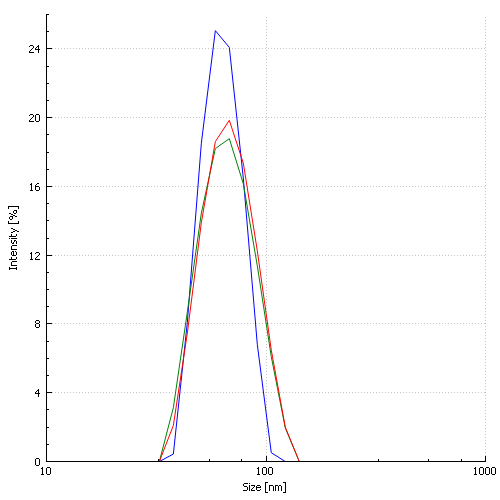 | 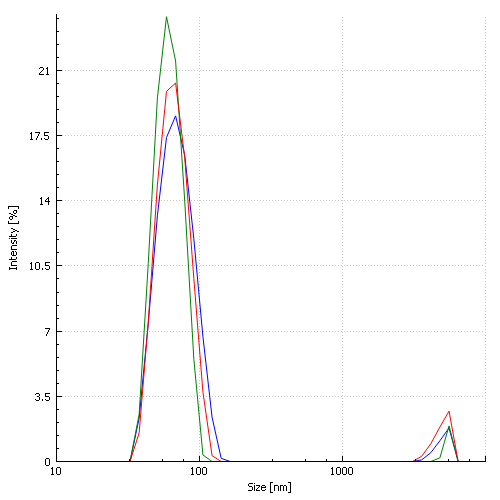 |
| --- | --- |
| Figure S.6.7. Size distribution of βBodipy FL-labelled liposome population with the dye to lipid molar ratio equals to 1:500. | Figure S.6.8. Size distribution of βBodipy FL-labelled liposome population with the dye to lipid molar ratio equals to 1:1000. |

Fluoresceine-PE; D=100nm

| 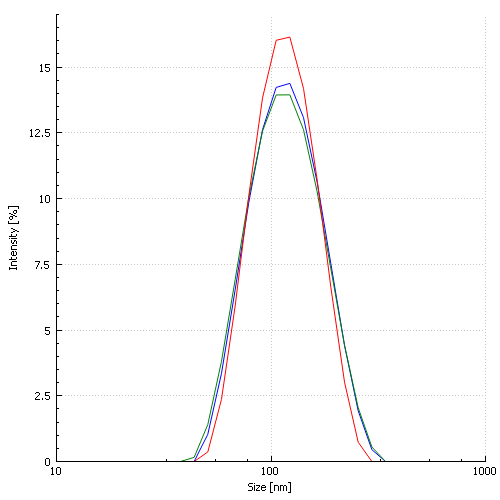 | 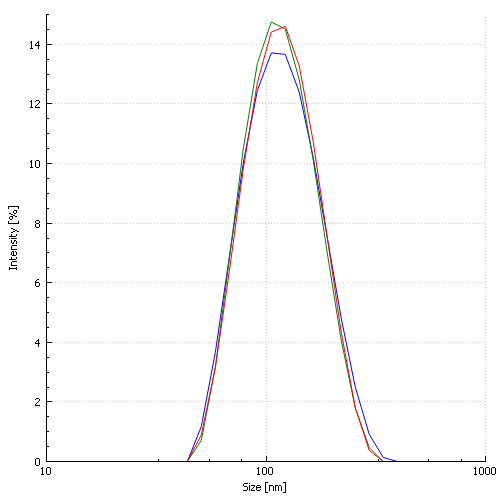 |
| --- | --- |
| Figure S.6.9. Size distribution of Fluoresceine-PE-labelled liposome population with the dye to lipid molar ratio equals to 1:500. | Figure S.6.10. Size distribution of Fluoresceine-PE-labelled liposome population with the dye to lipid molar ratio equals to 1:1000. |

Fluoresceine-PE; D=50nm

| 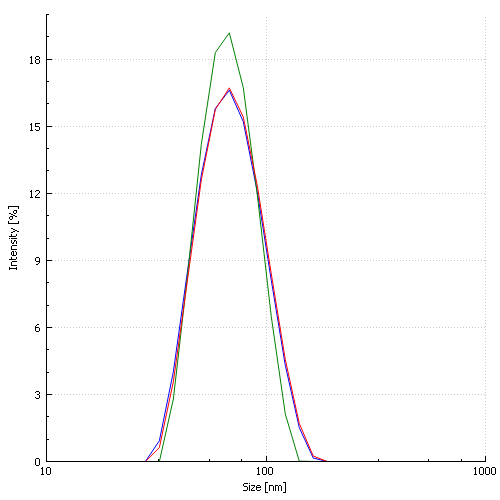 | 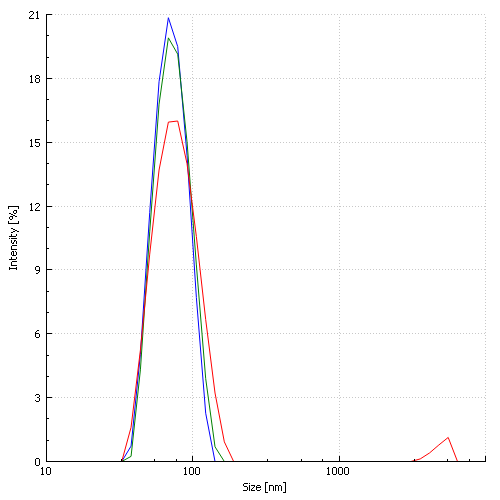 |
| --- | --- |
| Figure S.6.11. Size distribution of Fluoresceine-PE-labelled liposome population with the dye to lipid molar ratio equals to 1:500. | Figure S.6.12. Size distribution of Fluoresceine-PE-labelled liposome population with the dye to lipid molar ratio equals to 1:1000. |

NBD-PE; D=100nm

| 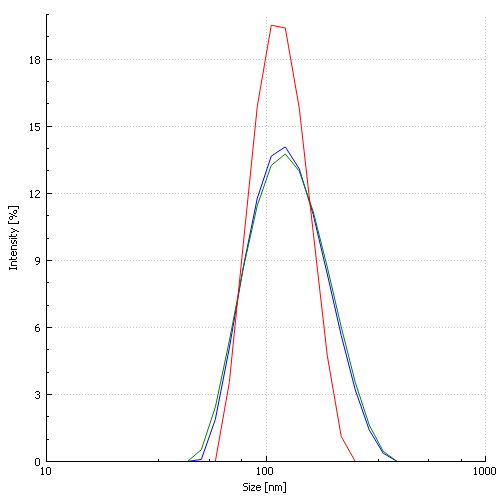 | 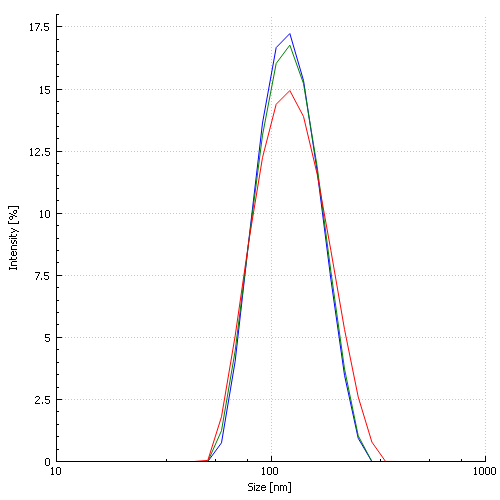 |
| --- | --- |
| Figure S.6.13. Size distribution of NBD-PE-labelled liposome population with the dye to lipid molar ratio equals to 1:500. | Figure S.6.14. Size dstribution of NBD-PE-labelled liposome population with the dye to lipid molar ratio equals to 1:1000. |

NBD-PE; D=50nm

| 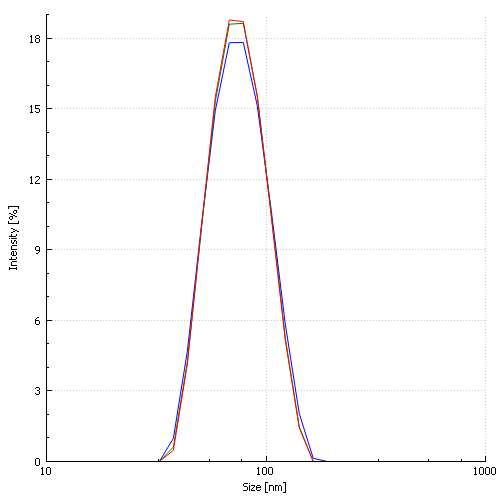 | 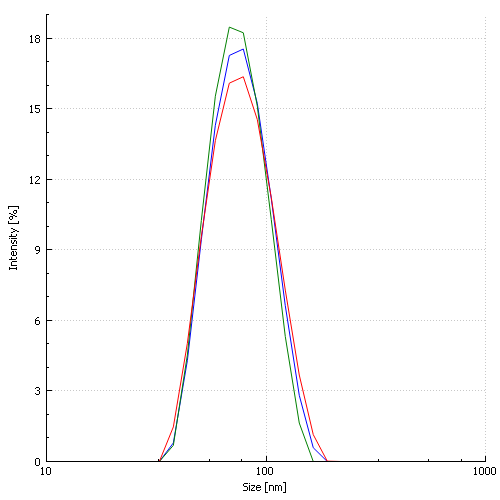 |
| --- | --- |
| Figure S.6.15. Size distribution of NBD-PE-labelled liposome population with the dye to lipid molar ratio equals to 1:500. | Figure S.6.16. Size distribution of NBD-PE-labelled liposome population with the dye to lipid molar ratio equals to 1:1000. |

# Effective focal volumes determinate by fitting Brownian and Liposome models to experimental data


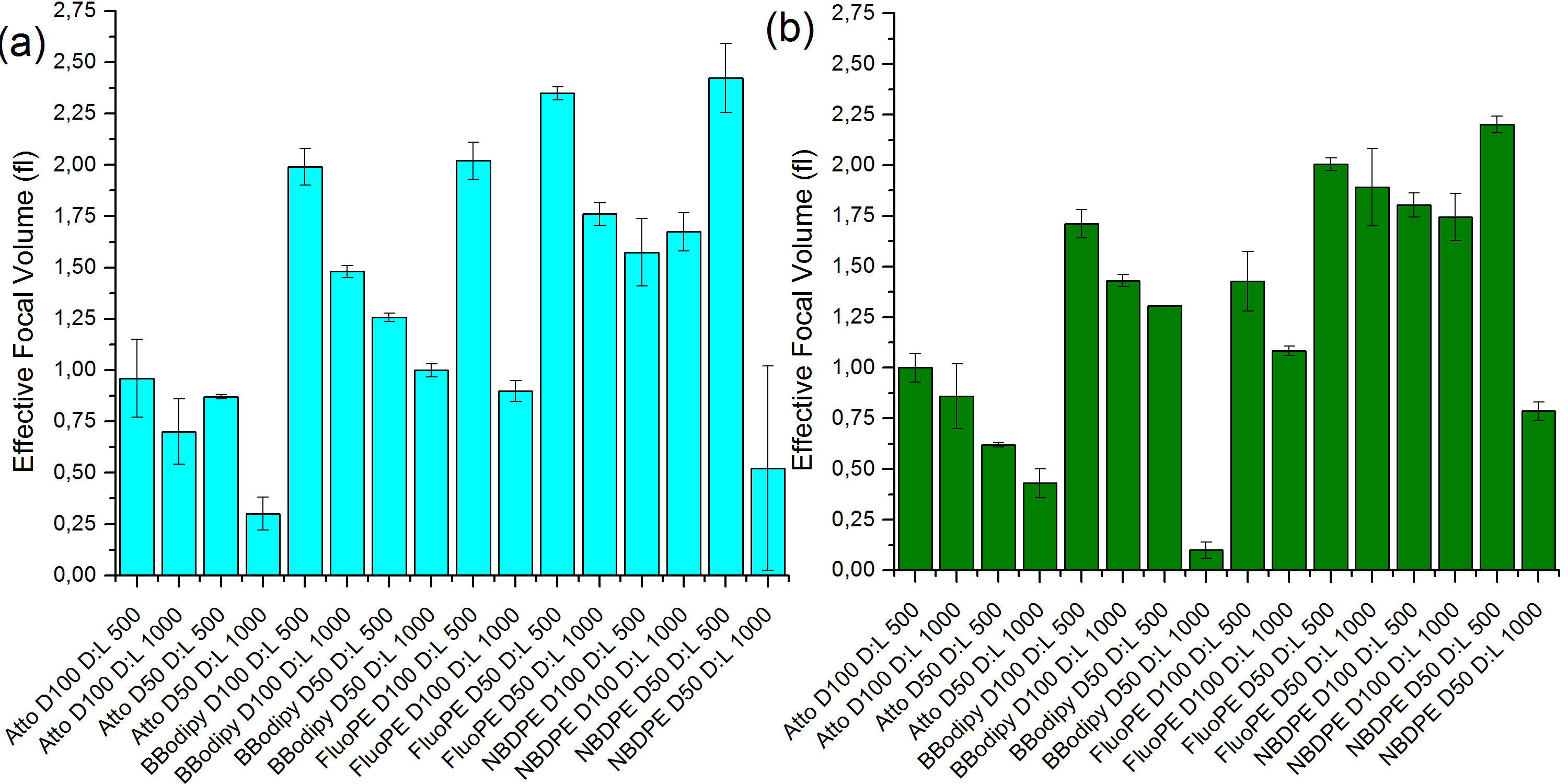


Figure S.7. Focal volumes calculated based on parameters acquired from DLS measurements assuming the Brownian Motion model and using (a) Levenberg-Marquardt or (b) Genetic algorithms, respectively.


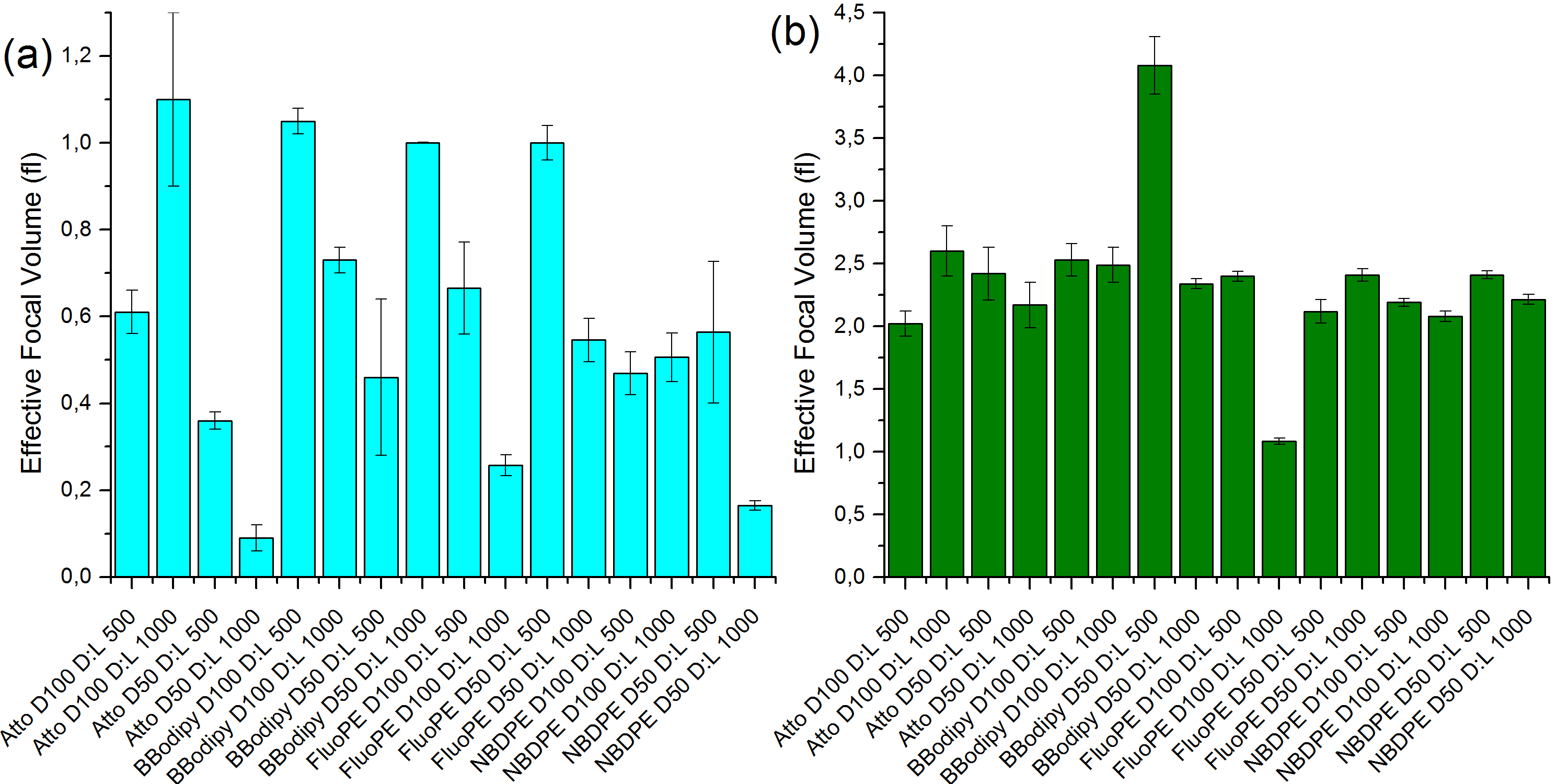


Figure S.8. The focal volumes calculated based on parameters acquired from DLS measurements assuming the Liposome model and using (a) Levenberg-Marquardt or (b) Genetic algorithms, respectively.
